# Multiple Origins of the Pathogenic Yeast *Candida orthopsilosis* by Separate Hybridizations between Two Parental Species

**DOI:** 10.1371/journal.pgen.1006404

**Published:** 2016-11-02

**Authors:** Markus S. Schröder, Kontxi Martinez de San Vicente, Tâmara H. R. Prandini, Stephen Hammel, Desmond G. Higgins, Eduardo Bagagli, Kenneth H. Wolfe, Geraldine Butler

**Affiliations:** 1 School of Biomedical and Biomolecular Science and UCD Conway Institute of Biomolecular and Biomedical Research, Conway Institute, University College Dublin, Belfield, Dublin, Ireland; 2 Instituto de Biociências, UNESP - Univ Estadual Paulista, Botucatu, Sao Paulo, Brazil; 3 School of Medicine and UCD Conway Institute of Biomolecular and Biomedical Research, University College Dublin, Belfield, Dublin, Ireland; Stanford University, UNITED STATES

## Abstract

Mating between different species produces hybrids that are usually asexual and stuck as diploids, but can also lead to the formation of new species. Here, we report the genome sequences of 27 isolates of the pathogenic yeast *Candida orthopsilosis*. We find that most isolates are diploid hybrids, products of mating between two unknown parental species (A and B) that are 5% divergent in sequence. Isolates vary greatly in the extent of homogenization between A and B, making their genomes a mosaic of highly heterozygous regions interspersed with homozygous regions. Separate phylogenetic analyses of SNPs in the A- and B-derived portions of the genome produces almost identical trees of the isolates with four major clades. However, the presence of two mutually exclusive genotype combinations at the mating type locus, and recombinant mitochondrial genomes diagnostic of inter-clade mating, shows that the species *C*. *orthopsilosis* does not have a single evolutionary origin but was created at least four times by separate interspecies hybridizations between parents A and B. Older hybrids have lost more heterozygosity. We also identify two isolates with homozygous genomes derived exclusively from parent A, which are pure non-hybrid strains. The parallel emergence of the same hybrid species from multiple independent hybridization events is common in plant evolution, but is much less documented in pathogenic fungi.

## Author Summary

The genus *Candida* is one of the leading causes of fungal morbidity in humans. Many pathogenic *Candida* species are diploid, and do not have have a full sexual cycle. The evolutionary origin of *Candida orthopsilosis* is unclear. Here, we use whole genome sequencing of 27 *C*. *orthopsilosis* isolates from around the world to show that *C*. *orthopsilosis* arose from hybridization (or mating) of two distinct parental species. Unusually, the hybridization event did not occur only once; we identify at least four events, and we suggest that hybridization is ongoing. The “species” *C*. *orthopsilosis* therefore does not have one single origin. We have identified one of the parental lineages involved, but the other remains elusive. Our results suggest that inter-species hybridization has an evolutionary advantage. However, unlike in plant pathogens, it does not appear to result in increased virulence of *C*. *orthopsilosis*.

## Introduction

Hybridization or mating between different species can promote the emergence of new species by creating extreme (transgressive) phenotypes allowing adaptation to new ecological niches [1]. In the human fungal pathogen *Cryptococcus neoformans*, hybridization has been associated with phenotypic evolution and increased virulence [2, 3], and in plant fungal pathogens hybridization is associated with increased host range and the emergence of new species [4–6]. Hybridization is particularly common in yeast species used in the preparation of food and drink, such as *Zygosaccharomyces* and *Saccharomyces* [7–9]. Natural hybrids between many of the members of the *Saccharomyces* species complex have been identified [10, 11]. For example, *Saccharomyces pastorianus* formed at least twice from recent hybridizations between *Saccharomyces cerevisiae* and *Saccharomyces eubayanus*, and this event has been associated with the acquisition of cold tolerance in the lager yeast [12–14].

Homoploid hybrid speciation (without an increase in chromosome number) can lead to the formation of new species, for example in natural populations of *Saccharomyces paradoxus* [15]. Polyploidization was probably important for speciation of up to 1/3 of plants, and has been reported in both plants and animals [16]. The increased use of whole genome sequencing has made it relatively easy to identify hybrids and to study their genome evolution at high resolution [9], and indeed recent evidence suggests that the whole-genome duplication in the *S*. *cerevisiae* lineage arose from an ancient hybridization between two closely related species [17].

Here, we investigate hybridization in members of the yeast CTG-Ser clade (species that translate the codon CTG as serine and not leucine [18]). Several of these species are human fungal pathogens, including *Candida parapsilosis*, which is particularly associated with infections of neonates [19–21]. The *C*. *parapsilosis sensu lato* species complex consists of three defined species: *C*. *parapsilosis sensu stricto*, *C*. *orthopsilosis* and *C*. *metapsilosis* [22]. *C*. *parapsilosis sensu stricto* is the most frequently isolated from human infections, followed by *C*. *orthopsilosis* (up to 26% of *C*. *parapsilosis sensu lato* isolates) and *C*. *metapsilosis* (up to 11% of *C*. *parapsilosis sensu lato* isolates) [23, 24]. There is however a large variation in the frequency of isolation of the individual species, which may be related to geographic region. Several studies fail to identify any *C*. *metapsilosis* isolates [23, 24], whereas in a 12-year study in Taiwan, approximately equal numbers (10%) of *C*. *parapsilosis sensu lato* isolates were identified as *C*. *orthopsilosis* and *C*. *metapsilosis* [25]. A recent study in Chinese hospitals identified more *C*. *metapsilosis* than *C*. *orthopsilosis* isolates [26]. The *C*. *parapsilosis sensu lato* species vary significantly in virulence and drug susceptibility, with *C*. *parapsilosis* being the most virulent, followed by *C*. *orthopsilosis* and *C*. *metapsilosis* [25, 27, 28].

*C*. *parapsilosis sensu lato* species are obligate diploids, and mating and meiosis have never been observed [29–31]. The level of heterozygosity in *C*. *parapsilosis sensu stricto* isolates is much lower than in other CTG clade species [29, 32–34]. For example, SNP frequency in one sequenced *C*. *parapsilosis* isolate is approximately 1 SNP per 15 kb, which is 70 times lower than in the related species *Lodderomyces elongisporus* [29]. Low levels of heterozygosity were confirmed by sequencing three additional genomes, though some copy number variations were identified [34]. In addition, all *C*. *parapsilosis sensu stricto* isolates characterized to date contain only one mating idiomorph (*MTL*a) at the Mating-Type Like locus, and *MTL*a1 is a pseudogene [31]. Genome structure in *C*. *metapsilosis* however suggests a different evolutionary history in that species. Sequencing genomes of 11 clinical *C*. *metapsilosis* isolates showed that they were all highly heterozygous, and most likely resulted from hybridization between two parental species that differed by approximately 4.5% at the genome level [35]. Although earlier analysis suggested that *C*. *metapsilosis* isolates contained only MTLα idiomorphs [31] genome sequencing revealed that a second idiomorph was formed by introgression at MTLa generating a chimeric locus, containing the MTLa regulatory genes a1 and a2, and MTLα2 [35]. The authors suggested that a single ancient interspecies hybridization event was followed by global expansion of *C*. *metapsilosis* and loss of heterozygosity [35]. In *C*. *orthopsilosis*, AFLP (amplification fragment length polymorphism) analysis and sequencing of ITS sequences identified some heterogeneity among isolates, suggesting the presence of at least two sub-groups [31, 36–38]. This was supported by our identification of two MTLa and two MTLα idiomorphs in 16 *C*. *orthopsilosis* isolates which differed by approximately 5% [31]. Some isolates were heterozygous at MTL, and we suggested that the two different MTLa/α combinations represented two distinct subspecies, named Type 1 and Type 2.

Sequencing of a putative *C*. *orthopsilosis* Type 2 genome (isolate 90–125) showed that it is highly homozygous, similar to *C*. *parapsilosis* [29, 39]. However, further studies identified two highly heterozygous isolates, which were suggested to result from the same hybridization event, possibly between Type 1 and Type 2 parents [40]. Here, we carried out a population genomics analysis of 27 worldwide *C*. *orthopsilosis* isolates. We report that most *C*. *orthopsilosis* isolates are hybrids most likely formed by mating between two parental species that are about 5% different in sequence, followed by loss of heterozygosity (LOH) to form mosaic genomes.

Although some aspects of *C*. *orthopsilosis* evolution are remarkably similar to the recently described structure of *C*. *metapsilosis* populations [35], we show that *C*. *orthopsilosis* has arisen from at least 4 distinct hybridizations between the same two parental species, whereas all known *C*. *metapsilosis* strains derive from a single ancestral hybridization. We propose a model for *C*. *orthopsilosis* hybrid origins that places Type 1 and Type 2 strains in the hybrid context, and shows that Type 1 and Type 2 are not useful descriptions of the parental species, but are reciprocal combinations of mating partners. The existence of recombinant mitochondrial genomes and a non-hybrid “Parent A” lineage indicates that the formation of *C*. *orthopsilosis* by hybridization is recent and probably ongoing. Recurrent hybridization has been shown to lead to increased virulence of some plant and animal fungal pathogens [2, 4, 41–44], but our study is the first to show that it is also occurring in the *Candida* clade.

## Results and Discussion

### Sequence analysis of 27 *C*. *orthopsilosis* strains

We sequenced the genomes of 27 *C*. *orthopsilosis* clinical isolates from around the globe, including Europe, US and Asia (Table 1). Four isolates were sequenced at >400X coverage with the reminder at >70X with Illumina technology. One isolate (Sample 427) was also sequenced using PacBio technology, which was used to characterize genome structure. Previously sequenced isolates 90–125 and MCO456 [39, 40] were included in the subsequent analysis. We identified an assembly error in the 90–125 reference genome, an artefactual translocation between chromosomes 2 and chromosome 6 (S1 Fig). The corrected assembly has been submitted to GenBank (BioProject number PRJEA83665). A description of the main findings from the genome data, including an analysis of copy number variation, is provided in S1 File and is summarised in Table 1.

**Table 1:** Overview of *C*. *orthopsilosis* isolates.

Sample	Common Name	Clade	Type	Origin	Source	Mapped Reads	Coverage [x]	Variants
423	CP85	1	Catheter	L’Aquila, Italy	(Tav)	9.9 M	120	337,980
434	CP289	1	-	NCPF, UK	(Tav)	9.9 M	121	341,090
1799	CAS09-1799	1	Blood	Atlanta, USA	CDC	11.1 M	135	334,763
^MCO456	MCO456	1	-	San Antonio, USA		68.8 M	544	324,303
425	CP125	2	Nail	Pisa, Italy	(Tav)	8.8 M	106	435,639
426	CP185	2	Blood	Varese, Italy	(Tav)	10.5 M	127	391,692
427	CP269	2	Br. asp.	Pisa, Italy	(Tav)	55.8 M	677	406,441
**427**	**CP269**	**2**	**Br. asp.**	**Pisa, Italy**		**0.2 M**	**47**	-
435	CP296	2	Skin	Pisa, Italy	(Tav)	12.2 M	148	400,473
436	CP331	2	Sputum	Pisa, Italy	(Tav)	11.1 M	134	412,291
504	CAS08-0504	2	Blood	Baltimore, USA	CDC	10.1 M	122	408,796
831	CAS09-0831	2	Blood	Atlanta, USA	CDC	45.1 M	552	402,495
B-8323	B-8323	2	-	Pakistan	CDC	5.8 M	71	427,257
151	CAS08-0151	3	Blood	Atlanta, USA	CDC	9.3 M	112	385,324
185	CAS08-0185	3	Blood	Atlanta, USA	CDC	9.4 M	113	373,387
421	CP25	3	Nail	Pisa, Italy	(Tav)	10.5 M	127	402,521
422	CP47	3	Skin	Pisa, Italy	(Tav)	39.5 M	478	370,184
433	CP288	3	Nail	St. Niklaas, Belgium	(Tav)	10.1 M	122	390,308
437	CP344	3	Catheter	Pisa, Italy	(Tav)	10.6 M	128	390,761
599	CAS08-0599	3	Blood	Baltimore, USA	CDC	10.6 M	129	388,277
1540	CAS09-1540	3	Blood	Baltimore, USA	CDC	11.3 M	136	390,050
1825	CAS10-1825	3	Blood	Baltimore, USA	CDC	12.4 M	151	399,895
B-8274	B-8274	3	-	Pakistan	CDC	10.2 M	123	390,810
424	CP124	4.1	Br. Asp.	Pisa, Italy	(Tav)	11.2 M	136	460,223
748	CAS09-0748	4.1	Blood	Atlanta, USA	CDC	11.2 M	138	468,132
282	CAS08-0282	4.2	Blood	Baltimore, USA	CDC	38.1 M	464	446,819
320	CAS08-0320	4.2	Blood	Baltimore, USA	CDC	65.1 M	792	436,695
498	CAS08-0498	4.3	Blood	Baltimore, USA	CDC	10.2 M	124	457,148
428	CP287	-	Toenail	Hong Kong	(Tav)	10.4 M	126	61,092
^90–125	90–125	-	-	San Francisco, USA	(Tav)	34.8 M	105	3,038

Br. Asp. = Bronchial aspirate. NCPF = The National Collection of Pathogenic Fungi.

Mapped reads, coverage and variants are compared to the 90–125 reference genome.

Variants include SNPs, insertions and deletions. Sample 427 was sequenced using Illumina and PacBio technologies; the PacBio data is highlighted in bold.

^The genome sequences of MCO456 and 90–125 were described previously [39, 40]. Tav = Tavanti et al (2007) [32].

### Most *C*. *orthopsilosis* isolates are hybrids

**Fig 1:**
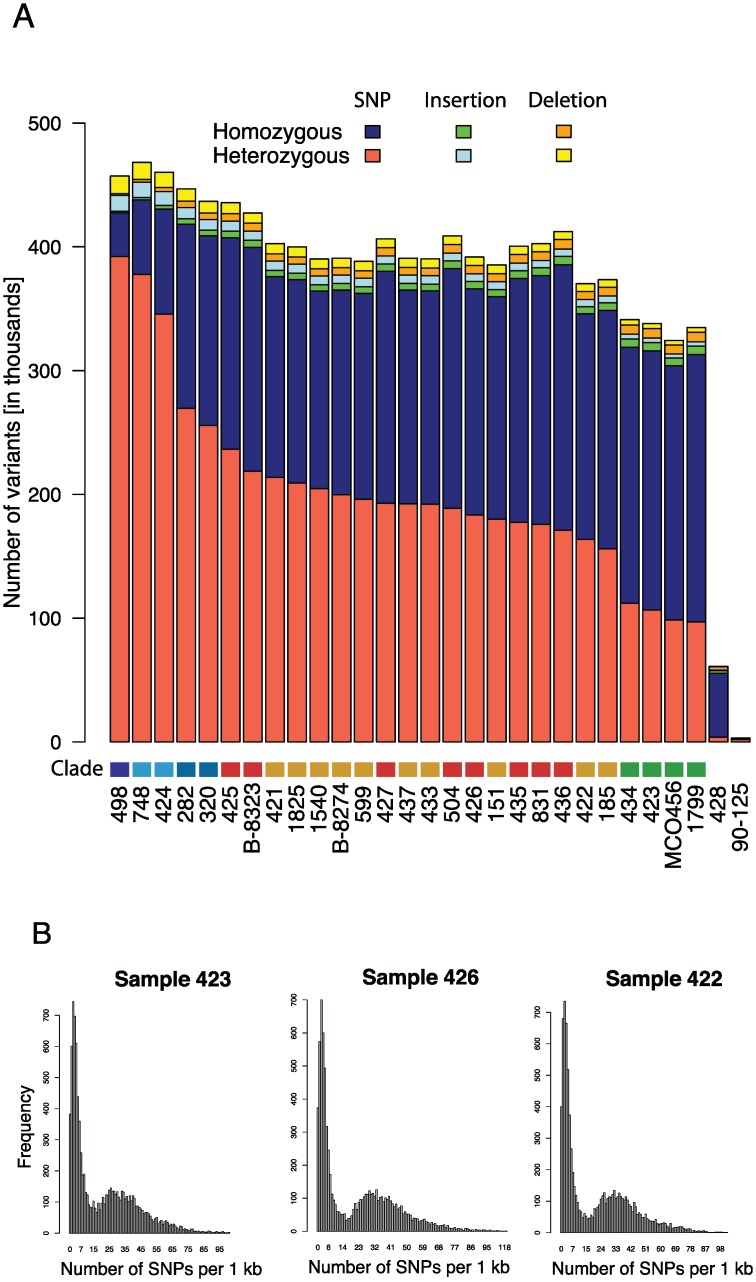
The majority of *C*. *orthopsilosis* strains are highly heterozygous. A. The total number of homozygous and heterozygous single nucleotide polymorphisms (SNPs, blue and red), insertions (green and light blue) and deletions (orange and yellow) across all sequenced isolates are shown relative to isolate 90–125. Strains are ordered left-to-right relative to the number of heterozygous SNPs. The “Clade” color refers to clades identified in Fig 2; Clade 1 (green), Clade 2 (red), Clade 3 (orange), Clade 4.1 (light blue), Clade 4.2 (blue) and Clade 4.3 (dark blue). B. The distribution of homozygous SNPs in 1 kb regions relative to isolate 90–125 is shown for one isolate from Clade 1 (Sample 423), Clade 2 (Sample 426) and Clade 3 (Sample 422). The SNP distribution is bimodal, with some regions almost identical to 90–125, and some regions that differ by >3%. When all regions are taken into account, the A and B haplotypes differ by 5.1%. These most likely represent the A and B haplotypes. The distribution patterns for all isolates are shown in S2 Fig.

Homozygous and heterozygous single nucleotide polymorphisms and insertions and deletions relative to the reference genome 90–125 were identified as described in Methods (Fig 1). Only one strain has a highly homozygous genome similar to 90–125 (Sample 428, <4,000 heterozygous SNPs, mostly derived from incorrectly assembled regions in the reference strain). In contrast, the heterozygosity levels of the remaining isolates varied from approximately 100,000 (Sample 1799) to 400,000 (Sample 498) heterozygous sites, and are much more similar in number to isolate MCO456 described by Pryszcz et al [40].

Pryszcz et al [40] suggested that high levels of heterozygosity in *C*. *orthopsilosis* MCO456 and in a related isolate AY2 reflects their origin from a hybridization between two related, but different, species or sub-species, where one parent is highly similar to 90–125. This proposal was supported by their observation that there is a bimodal distribution of differences between homozygous regions of MCO456 and 90–125. Approximately 41% of the MCO456 genome is similar to 90–125 (<1.8% divergence), and approximately 32% is different (>1.8% divergence). We carried out a similar analysis of our data, and we found that all the heterozygous isolates exhibited a similarly bimodal pattern of differences to 90–125 (Fig 1B, S2 Fig). This result indicates that the majority of *C*. *orthopsilosis* isolates arose from hybridization between two parents. The average nucleotide difference is 5.1%. One of the parents is very similar to 90–125.

**Fig 2:**
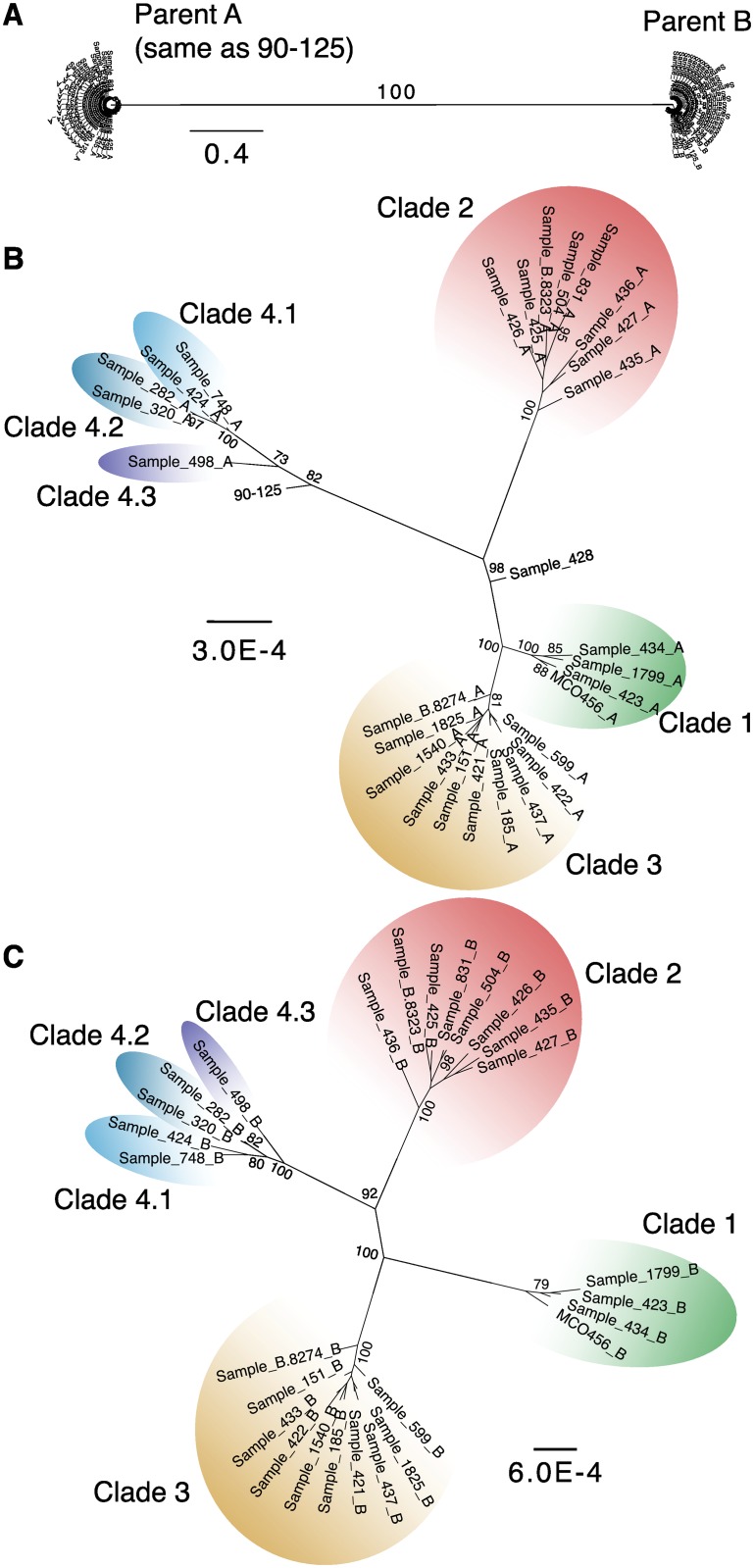
*C*. *orthopsilosis* isolates belong to several clades. A. Maximum likelihood (ML) trees of SNPs extracted from 1 kb regions that are heterozygous in at least 20 isolates (excluding homozygous strains 90–125 and Sample 428). We included homozygous and heterozygous SNPs from each 1 kb region and assigned one of the heterozygous bases as haplotype A (parental species A, PSA) if it was the same as 90–125, and the other base to haplotype B (PSB). Homozygous SNPs not present in 90–125 were assigned to haplotype B. B. ML tree using only haplotype A bases. C. ML tree using only haplotype B bases. Clades are colored as green (Clade 1), red (Clade 2), orange (Clade 3), light blue (Clade 4.1), blue (Clade 4.2), and dark blue (Clade 4.3). Node labels indicate bootstrap values from 1000 replicates. Only bootstrap support values greater than 70% are shown.

To further characterize the parental lineages, we identified the regions of each genome derived from each parent, and used these in a phylogenetic reconstruction. We first identified highly heterozygous regions (defined as 1 kb regions that were heterozygous in at least 20 isolates, see Methods). We then identified the homozygous and heterozygous SNPs from these regions and assigned them to haplotypes. Those that were identical to isolate 90–125 were assigned to haplotype A, and those that were different were assigned to haplotype B. Maximum likelihood trees were generated separately from each haplotype using RAxML [45] (Fig 2). When all SNPs are considered together, the isolates fall into only two groups (Fig 2A), suggesting that they all arose from the same two parents, where haplotype A is derived from one parental species, and haplotype B from the other.

The phylogenetic trees derived independently from SNPs in each of the two haplotypes are very similar (Fig 2). The isolates fall into 4 major clades, with the two homozygous isolates forming outgroups to Clades 1 and 3 (Sample 428) and Clade 4 (90–125). Clade 4 is somewhat divergent, and for discussion we divide it into sub-clades 4.1 and 4.2, with a single isolate (Sample 498) in subclade 4.3. The overall sequence divergence in the trees for haplotype B is almost twice as large as for haplotype A (indicated by scale in Fig 2).

**Fig 3:**
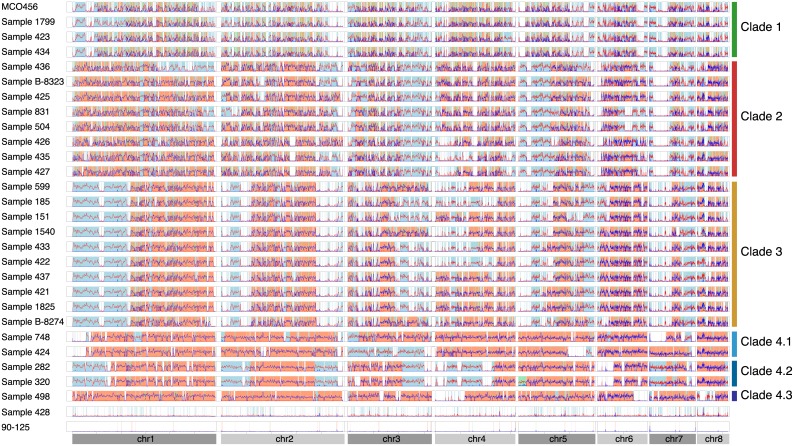
Loss of heterozygosity (LOH) events in *C*. *orthopsilosis* isolates. The distribution of homozygous (red) and heterozygous (blue) SNPs in 1 kb windows for all isolates compared to the reference sequence of isolate 90–125 is shown. Each row represents a different strain, with chromosomes ordered from left to right. Lines are smoothed using Loess fit in R (smoothing parameter alpha = 0.01). The background colors indicate regions with LOH: homozygous A (derived from PSA only) is shown in white, homozygous B (derived from PSB only) is shown in blue. Heterozygous A/B regions are shown in orange and regions that cannot be assigned to A or B (undefined) are shown in green. The small number of SNPs identified in the 90–125 reads results from errors in the original assembly. Clades are defined in Fig 2.

We next assigned all sections of each genome (in 1 kb windows) into one of four categories: homozygous haplotype A, homozygous haplotype B, heterozygous (AB), and undefined, based on the distribution of homozygous and heterozygous SNPs relative to 90–125 (Fig 3, see Methods). Loss of heterozygosity (LOH) events (homozygous A or homozygous B) are shared by members of the same clade, though some events are specific to individual isolates within each clade. Clade 1 isolates have the highest amount of LOH, followed by isolates in Clade 2, 3 and 4. The majority of large LOH events occurred towards telomeric regions, particularly in the most heterozygous isolates in Clade 4.

To test whether A and B are the only two haplotypes present in the sequenced *C*. *orthopsilosis* isolates, we investigated whether there is any evidence for genomic regions that originate from a third source. To do this, we used the 90–125 strain as a reference for haplotype A, and inferred an almost-complete (84.6%) reference for haplotype B from sample 424, which has the lowest amount of homozygous haplotype A regions. We then repeated the SNP analysis looking for genomic regions that are divergent from both the A and B references (S3 Fig). The majority of the genomes are similar to either haplotype A or haplotype B, except for regions where haplotype B cannot be inferred (S3 Fig). Some short regions on chromosomes 7 and 8 in some isolates differ from both haplotypes A and B, but this is an artifact of short LOH events (< 500 bp) in sample 424 (S3 Fig). We therefore conclude that all heterozygous isolates descended from the same two parental species, A and B.

**Fig 4:**
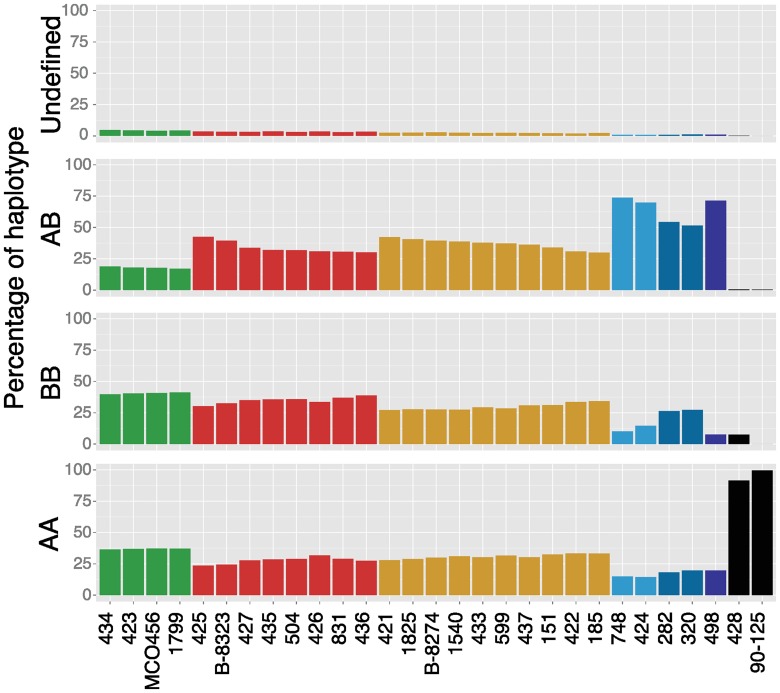
There is no bias towards haplotype A or haplotype B during LOH events in heterozygous isolates. The percentages of 1 kb regions of each isolate categorized as homozygous haplotype A (AA), homozygous haplotype B (BB), heterozygous (AB) and undefined are shown. Isolates are ordered according to their clade (Fig 2; Clade 1 green, Clade 2 red, Clade 3 orange, Clade 4.1 light blue, Clade 4.2 blue and Clade 4.3 dark blue) and by the amount of heterozygosity within each clade.

For the heterozygous isolates we determined the contribution from each parent by calculating the percentage of each strain’s genome that is homozygous AA, homozygous BB, heterozygous AB or undefined (Fig 4, see Methods). Most isolates have approximately equal contributions from haplotype A and haplotype B genome-wide. Isolates with the biggest difference include Sample 498 (12% more haplotype A than B) and Sample 436 (11% more haplotype B than A). Overall however, LOH events appear mostly random in *C*. *orthopsilosis* isolates with little preference towards one or the other parental species.

To determine if haplotypes A and B underwent recombination we took advantage of the PacBio data from Sample 427. We restricted the analysis to five regions where two haplotypes were assembled, ranging in size from 20 kb to 64 kb. In all five cases one contig from the PacBio assembly matched 90–125 (the A genome), and the other represented the B parent, indicating that no recombination occurred, at least at these regions in Sample 427 relative to 90–125 (S1 Table, S4 Fig).

### MTL genotypes show that *C*. *orthopsilosis* isolates arose from more than one hybridization event

We previously characterized the Mating-Type-Like (MTL) locus of 16 isolates of *C*. *orthopsilosis* and showed that both the MTLa and MTLα idiomorphs occurred in two types [31]. The types diverged in sequence by approximately 5%, for both MTLa and MTLα. The majority of isolates in that study were homozygous for either MTLa or MTLα and only two were MTLa/α heterozygotes. In that study, we assumed that these MTL heterozygotes resulted from mating between isolates in a single type or group; the MTLa and MTLα idiomorphs from isolate J981224 were designated as Type 1, and those from isolate CP125 (Sample 425) as Type 2. The 5% sequence divergence between the Type 1 and Type 2 MTL idiomorphs is similar to what we now observe between haplotypes A and B at other loci.

We analyzed the MTL idiomorphs in our genome sequences (which included 14 of the strains studied by Sai et al [31]). Six isolates are MTLa/α heterozygotes (S5 Fig). Analysis of adjacent SNPs shows that MTLα Type 1 and MTLa Type 2 are both in physical linkage with haplotype A in different strains (S5 Fig). Similarly, MTLα Type 2 and MTLa Type 1 are both linked to haplotype B. Isolates that are homozygous at MTL (either MTLa/a or MTLα/α) have undergone LOH, and SNP analysis shows that these tracts of LOH extend into the regions flanking the MTL locus itself so that both chromosomes are derived from the same parental haplotype (e.g. Samples 423 and 426 are MTLα/α homozygotes derived from haplotypes A and B respectively by LOH; S5 Fig). Some of the isolates heterozygous at MTL show a small amount of LOH in a region to the right of the MTL (Sample 425; S5 Fig).

We can now see that the MTLa/α heterozygotes designated “Type 1” by Sai et al [31] (such as Sample 498) are heterozygotes containing MTLα from haplotype A and MTLa from haplotype B. The heterozygotes that were designated as “Type 2” (such as Sample 425) have the reciprocal combination of MTLa from haplotype A and MTLα from haplotype B. Therefore, the “Type 1” and “Type 2” labels for MTL heterozygotes represent the two complementary ways that cells from two putative parental species corresponding to haplotypes A and B could combine by mating. We refer to these parental species as ‘Parental Species A’ and ‘Parental Species B’. Although many *Candida* species are asexual, a parasexual cycle has been described in some diploid species [46]. Cells of opposite mating type hybridize to form a tetraploid, followed by concerted chromosome loss to regenerate a diploid [47, 48]. We propose that similar events occurred during hybridization of Parental Species A and Parental Species B during the evolution of *C*. *orthopsilosis*, though we cannot rule out the possibility that the ancestral species were fully sexual.

**Fig 5:**
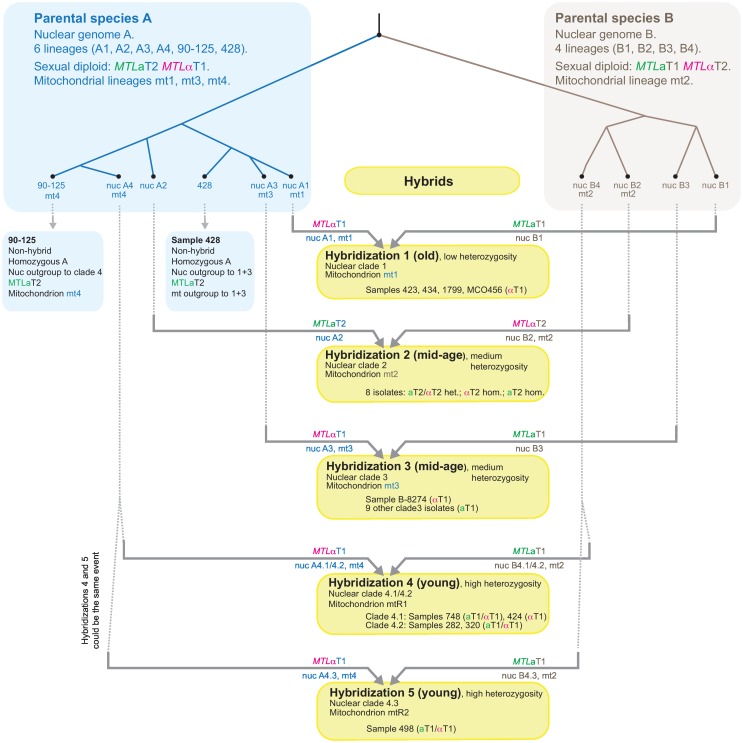
Multiple origins of the species *Candida orthopsilosis* by independent hybridization events. Each hybridization involved mating between cells of the same two parental species, A and B, which are 5% different in genome sequence. We propose that within species A and B, there are multiple distinct lineages. In species B we identify four lineages corresponding to the nuclear haplotypes B1-B4 found in hybrids. In species A we identify six lineages comprising the four nuclear haplotypes A1-A4 found in hybrids, and two found in the non-hybrid strains 90–125 and 428. The inferred mitochondrial (mt) mitotypes of each parental lineage are shown where known. The five yellow boxes represent the five separate hybridization events we propose, with the genomic contributions from parents A and B indicated on the horizontal arrows (mitotypes, nuclear haplotypes, and MTL genotypes). See text for details of the proposed hybridization events.

For isolates in Clades 1, 3 and 4, Parental Species A contributed MTLα, and Parental Species B contributed MTLa. Conversely, for Clade 2 isolates, Parental Species A contributed MTLa, and Parental Species B contributed MTLα (S5 Fig; Fig 5). This discrepancy in MTL genotypes indicates that Clade 2 and Clades 1/3/4 cannot be descendants of a single ancestral mating event between two cells from the two parental species, so at least two separate hybridization events occurred. By analysis of other datasets described below, we inferred a model for *C*. *orthopsilosis* evolution that postulates at least 4, and possibly 5 separate hybridizations between the parental species (Fig 5).

### Recombination of mitochondrial genomes

**Fig 6:**
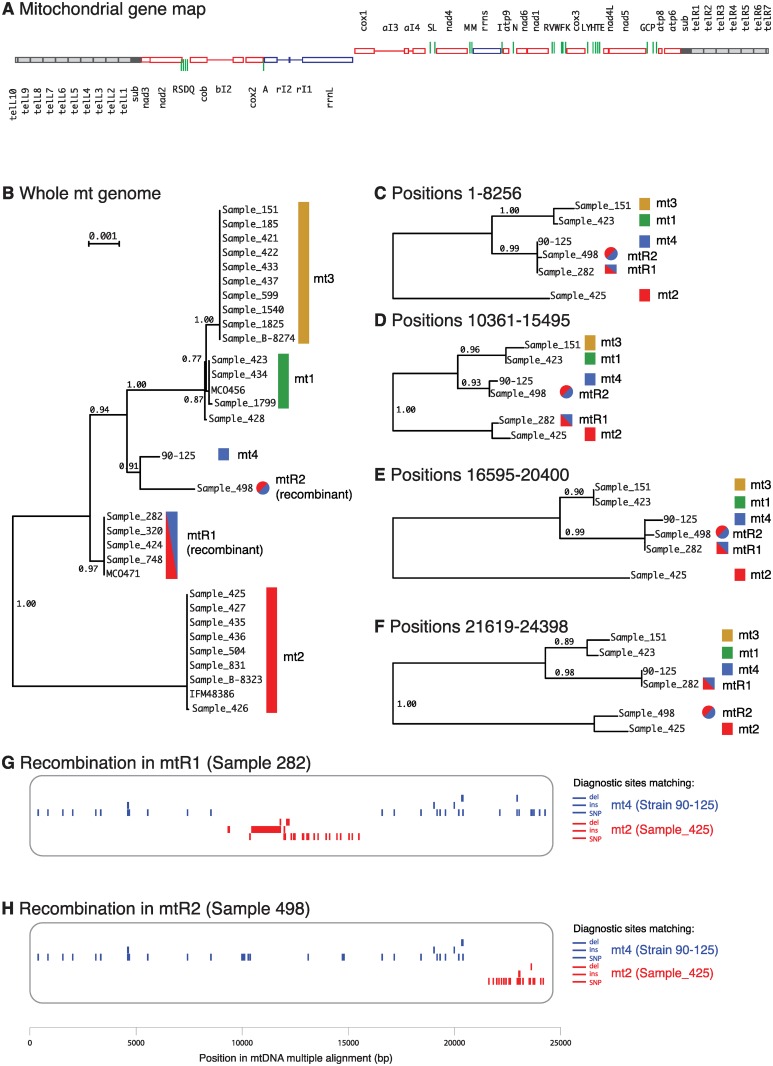
Phylogeny and recombination of *C*. *orthopsilosis* mitochondrial genomes. A. Gene map of the PacBio assembly of the mitochondrial genome from Sample 427 (33,782 bp). Subterminal repeats are black and telomeric repeats are grey. All genes from *cox1* to the right telomere are transcribed rightwards, and all genes from *rrnL* to the left telomere are transcribed leftwards. Thin horizontal lines represent introns. Green vertical lines are tRNA genes. B. Phylogenetic tree of the whole mitochondrial genome (excluding subterminal and telomeric regions) from all *C*. *orthopsilosis* strains, generated by PhyML from a Clustal Omega alignment in Seaview. (C–F). Phylogenetic trees of four sections of the genome, for strains representing mitochondrial haplotypes mt1-mt4 and recombinants. (G, H) Inference of recombination sites in recombinant mtDNAs. For each mitochondrial haplotype mt1-mt4 we identified SNPs, insertions and deletions that are unique to that haplotype (not shared with the other three). We then scanned the putatively recombinant strains Sample 282 (mtR1) and Sample 498 (mtR2) for the presence of these unique sites. Neither of these strains shares any unique sites with mt1 or mt3. We then repeated the analysis, defining a larger set of sites that are diagnostic for mt2 (not shared with mt4) or diagnostic for mt4 (not shared with mt2).

We assembled the mitochondrial genome sequences from the 27 sequenced isolates and from strain 90–125 [39], and compared them to the three previously published *C*. *orthopsilosis* mtDNAs [49, 50]. Twenty-eight isolates have linear mitochondrial genomes, and three have circular genomes (S2 Table). In the linear genomes, all the genes are located in a central 24 kb region, which is flanked on each side by a large inverted terminal repeat (Fig 6A). This repeat consists of a subterminal region (*sub*) followed by multiple tandem copies of a telomeric repeat (*tel*). The PacBio assembly of mtDNA from Sample 427 has nine complete copies of *tel* on the left arm, and six on the right, followed by an incomplete copy on both arms (100 to 103 bp). *C*. *parapsilosis* mtDNA molecules, which have a similar organization, have been reported to contain up to eight telomeric repeats on each arm [51].

The circular genomes in Samples 436 and 90–125 are caused by recombination at microhomologies in the subterminal repeats, similar to those previously described [49]. Although the Illumina assemblies of the linear mtDNAs are incomplete in the telomeric regions, there are at least two different types of subterminal region (451 and 396 bp) and two types of telomeric repeat (565 and 777 bp) in *C*. *orthopsilosis* (S2 Table).

The phylogenetic analysis showed that the *C*. *orthopsilosis* mitochondrial genomes belong to four mitotypes (Fig 6B). Isolates from three mitotypes (mt1, mt2 and mt3) correspond to the nuclear Clades 1, 2 and 3. We designated strain 90–125 as mitotype mt4 because this strain is closely related to nuclear Clade 4 and its mtDNA appears not to be recombinant (see below). There is almost no variation within the mitotypes (0–6 SNPs in the whole genome) whereas divergence between the mitotypes is significant (up to 222 nucleotide substitutions or 1.1% between mt2 and mt3).

In contrast to Clades 1–3, nuclear Clade 4 isolates have mtDNAs that fall at two distinct positions on the tree. Most belong to a single clade designated as clade mtR1, but Sample 498 forms a distinct lineage (mtR2) that is more closely related to mt4 (90–125) and to the mt1 and mt3 clades (Fig 6B).

The predicted phylogenetic relationship of mt1, mt2, mt3 and mt4 isolates is the same, irrespective of which region of the mtDNA is used for the phylogenetic analysis. However, the placement of the mtR1 and mtR2 isolates varies when different mtDNA regions are used to construct trees (Fig 6C–6F). This suggests that the mtDNA in mtR1 and mtR2 resulted from an inter-lineage recombination, as was previously proposed by Valach et al [50] for the strain MCO471 (mtR1). The mtR1 and mtR2 mtDNAs are both derived from recombination between the mt2 and mt4 mitotypes, but they were formed by two separate events because the recombinations occurred at different sites in the genome (Fig 6G and 6H).

Analysis of diagnostic SNP sites in mtDNA (Fig 6G) shows that in mtR1 the center of the mitochondrial genome (between *rrnL* and *rrnS*) comes from mt2 whereas both arms come from mt4. In contrast, in mtR2 only the right arm (from *nad5* to the telomere) comes from mt2. The left recombination event in mtR1 can be mapped to within the *rrnL* gene for the large subunit rRNA, because mtR1 shares a 44 bp insert at the 5’ end of *rrnL* with mt2, but lacks the rI1 and rI2 introns that are present at the 3’ end of *rrnL* only in mt2. A further polymorphism in *C*. *orthopsilosis* mtDNA concerns the *cox1* intron ai3, which is present only in the mt2, mt3 and mtR1 clades (S2 Table).

### At least 4 distinct origins of *C*. *orthopsilosis* by hybridizations

Analysis of the nuclear polymorphisms, MTL loci and mitochondrial genomes together shows that there were at least four independent hybridizations between Parental Species A and B (Fig 5). We infer that Parental Species A and B were both quite diverse, with multiple populations having distinct nuclear lineages (lineages B1 to B4 within Parental Species B; and lineages A1 to A4, 90–125 and 428 within Parental Species A) and distinct mitochondrial lineages (mt1-mt4). Each hybridization was a mating event that gave rise to one of the four nuclear clades. After each clade was formed by hybridization, it diversified (probably clonally, undergoing LOH), resulting in congruent SNP trees for its A and B subgenomes.

#### Hybridization 1

Nuclear Clade 1 includes the hybrids with the highest LOH (Samples 423, 434, 1799 and MCO456; Fig 1), suggesting that they result from an old hybridization. We propose that members of this clade are descendants of an ancient mating between cells with nuclear lineages A1 (from Parental Species A) and B1 (from Parental Species B) (Fig 5). The mitochondrial genome (mt1) in this hybrid likely originated from Parental Species A, because it is more closely related to mitochondrial genomes in other A-lineages such as mt4.

#### Hybridizations 2 and 3

Isolates in nuclear Clades 2 and 3 have medium levels of heterozygosity (Fig 1), suggesting that they are younger hybrids than Clade 1 isolates. However, their *MTL* loci were derived from different parents (S5 Fig), indicating that Clades 2 and 3 must have arisen from two separate mating events. The mitochondrial genomes of Clades 2 and 3 also have opposite sources: Clade 2 received mt2 from its Parental Species B parent (nuclear haplotype B2), whereas Clade 3 received mt3 from its Parental Species A parent (nuclear haplotype A3).

#### Hybridization 4

Isolates in Clade 4 (Samples 748, 424, 282, 320 and 498) have the highest heterozygosity of the nuclear genome (Fig 1). The first four of these (Clades 4.1 and 4.2) also have the recombinant mitochondrial genome mtR1, which is a recombinant between mt4 (from Parental Species A) and mt2 (from Parental Species B). A likely explanation is that these four isolates are descendants from a relatively recent mating between a lineage of Parental Species A (with nuclear haplotype A4, mitotype mt4) and a lineage of Parental Species B (with nuclear haplotype B4, mitotype mt2).

#### Hybridization 5

It is unclear whether Sample 498 (the sole member of Clade 4.3) is also a product of Hybridization 4, or a product of a separate mating event between parents very similar to those involved in Hybridization 4. Sample 498 differs from the Clade 4.1/4.2 isolates by having different sites of mitochondrial genome recombination (mtR2 versus mtR1). If hybridizations 4 and 5 were the same mating event, then the hybrid must have maintained both mt2 and mt4 mitochondrial genomes for some time while Clade 4 diversified, before resolution through independent recombination events.

The two homozygous isolates 90–125 and Sample 428 are representatives of Parental Species A, but they probably did not contribute directly to hybridization events. Their positions in the phylogenetic trees suggest that they are Parental Species A lineages that are sister to the lineages that were involved in interspecies mating events. In both the nuclear and mitochondrial trees, Sample 428 appears as an outgroup to Clades 1 and 3, and 90–125 is an outgroup to Clade 4 (Figs 2 and 6). The two homozygous isolates do not appear to be hybrids that subsequently lost all of haplotype B, because if that was the case these isolates would be more likely to lie within (rather than sister to) hybrid clades in the SNP tree of A-haplotypes. We did not find any isolates with only a B haplotype, probably because we do not know what ecological niche these isolates inhabit.

### Analysis of virulence

**Fig 7:**
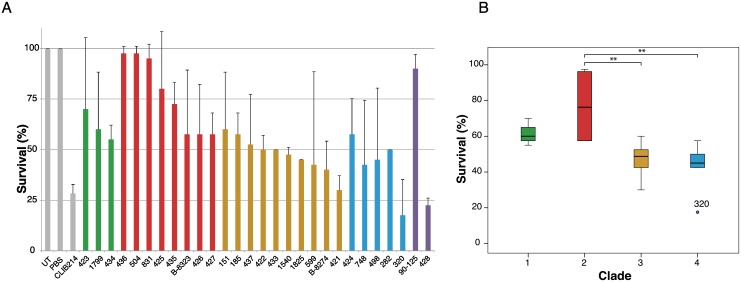
Virulence of *C*. *orthopsilosis* isolates. A. The percentage survival of *Galleria mellonella* 96 h after injection with 5 x 10^5^ cells of *C*. *orthopsilosis* strains (or 1 x 10^6^ for *C*. *parapsilosis* CLIB214) is shown. The survival assays were carried out twice, using 20 larvae per experiment. The graph shows the average and standard deviation of the percent surviving larvae at the endpoints from the two independent experiments. Columns from left to right represent: untreated control (UT), phosphate-buffered saline injected larvae (PBS), *C*. *parapsilosis* CLIB214, isolates from Clade 1 (green bars), Clade 2 (red), Clade 3 (orange), Clade 4 (blue), homozygous isolates 90–125 and 428 (purple). B. The endpoint survival rates between the clades were compared using ANOVA. When the two experiments are combined (i.e. 40 larvae infected with each isolate), Bonferroni multiple comparison correction showed that there is significant difference in survival between the strains in Clades 2 and 3 (p = 0.001) and between Clades 2 and 4 (p = 0.001). Considering each experiment individually, the p-values are marginally significant; p = 0.003 and 0.075 for Clade 2 versus 3, and p = 0.004 and 0.095 for Clade 2 versus 4.

Because hybridization has been associated with increased virulence of the human fungal pathogen *Cryptococcus neoformans* [2] and has been postulated as a virulence mechanism in *C*. *metapsilosis* [35], we measured the virulence of *C*. *orthopsilosis* isolates using the model host *Galleria mellonella* (Fig 7). We identified substantial variation, ranging from avirulent to highly virulent isolates (Fig 7A). Isolates in Clade 2 have significantly reduced virulence compared to isolates in Clade 3 and Clade 4 (Fig 7B). However, we did not identify any correlation between levels of heterozygosity and virulence, or between heterozygosity and doubling time in rich media, either by comparing survival endpoints or by using Kaplan-Meier analysis (Fig 7, S6 Fig). Notably, one homozygous *C*. *orthopsilosis* isolate (Sample 428) is virulent (survival rate <25%), whereas the other (90–125) is not (Fig 7, S6 Fig).

### Conclusion

All the isolates studied here would be classified as *C*. *orthopsilosis* based on their ribosomal DNA sequences, but our population genomics data shows that the name ‘*C*. *orthopsilosis*’ has been applied to two quite different types of isolates. A minority (2 of the 29 studied here) have ‘pure’ genomes that are simply Parental Species A. The first *C*. *orthopsilosis* genome sequenced, strain 90–125 [39] was fortuitously a strain of this type. In contrast, the majority of *C*. *orthopsilosis* isolates (93%; 27 of 29) are hybrids formed by mating between this species and a second species (Parental Species B) that is 5% different in genome sequence and that has not yet been isolated in a ‘pure’ (non-hybrid) form. This relationship is reminiscent of the beer yeasts, where a minority of strains are pure *S*. *cerevisiae*, the majority are interspecies hybrids (*S*. *cerevisiae* x *S*. *eubayanus*), and for a long time there was no known example of a ‘pure’ lineage of the *S*. *eubayanus* parent [12].

Our data show unequivocally that hybridization between the A and B parents of *C*. *orthopsilosis* occurred by mating, and that it occurred on at least four separate occasions. Since the pure A lineage was found in only 7% of isolates, and the pure B lineage was not found at all, this observation suggests that the interspecies hybrids are significantly more successful (i.e., more viable or more virulent) than their parents. Hybridization has been proposed to increase the virulence capacity of human fungal pathogens [2, 35] and the host range of plant fungal pathogens [4, 42]. However, our assays show no consistent correlation between virulence and heterozygosity in *C*. *orthopsilosis*, and a large difference in virulence between the two homozygous representatives of lineage A. Our results also indicate that *C*. *orthopsilosis* must be capable of mating, even though it has never been seen to mate in the laboratory.

The inference that multiple hybridizations occurred means that the species *C*. *orthopsilosis* does not have a single clonal origin. Although hybridization is common in yeasts, to our knowledge the only other known example of an ascomycete yeast species with multiple origins by parallel hybridizations is the beer yeast *S*. *pastorianus*. Multiple interspecies hybridizations have been reported in other pathogenic fungi (such as hybridizations between *Cryptococcus neoformans* and *C*. *deneoformans*, or between *C*. *neoformans* and *C*. *gattii*), but in these examples the hybrid lineages are a minority and the parental species are abundant and readily identifiable [2, 41]. We suggest that, for both *C*. *orthopsilosis* and *C*. *metapsilosis*, the parental species of these hybrids may be yeasts that are pathogens of other mammalian species and are not normally (or frequently) associated with humans. Formation of the hybrid may have facilitated a change in host range and pathogenicity to humans [2, 4, 35, 42]. Further investigation into geographical and ecological variation in the *C*. *parapsilosis sensu stricto* clade will be needed to understand the circumstances in which the parental species encounter one another and these pathogenic hybrids can emerge.

## Materials and Methods

### Strains and growth conditions

For Illumina sequencing, the 27 *C*. *orthopsilosis* isolates were cultured overnight in a shaking incubator at 30°C in 5 ml YPD medium (1% yeast extract, 2% peptone, 2% glucose). Genomic DNA was extracted using the Qiagen Genomic Tip-kit (20/G, product code 10223).

For PacBio sequencing, Sample 427 was grown in 100 ml synthetic complete media (2% glucose, 6.7% yeast nitrogen base, 2% Bacto agar, 0.2% dropout mix) overnight to reduce the carbohydrate concentrations. DNA was extracted using the Qiagen Genomic-tip kit (500/G, product code 10262) using a modified protocol for yeast (available at PacBio SampleNet, www.pacbiosamplenet.com).

### *Galleria mellonella* based virulence screen

*C*. *orthopsilosis* strains (Table 1) and *C*. *parapsilosis* CLIB214 were grown overnight in a shaking incubator at 30°C in 5 ml YPD medium (1% yeast extract, 2% peptone, 2% glucose). Yeast strains from overnight cultures were centrifuged and washed in Phosphate Buffered Saline (PBS, Oxoid) and diluted to 10^8^ cells/ml for *C*. *parapsilosis*, and 5 × 10^7^ cells/ml for *C*. *orthopsilosis* strains in PBS. *G*. *mellonella* in their final larval stage were obtained from Lifefoods Direct Ltd, Sheffield, UK, and stored at 15°C in the dark for use within 7 days from shipment. Twenty larvae, similar in size and weight, were used to analyze the virulence of each fungal strain, in two separate experiments. Larvae were injected with 10 μl of the diluted strains through the last left proleg, using insulin syringes. Untreated larvae and larvae injected with PBS were used as negative controls to assess the general viability and the effect of injection, respectively. After inoculation, larvae were placed into two petri dishes with filter paper (10 larvae per dish) and incubated at 30°C in the dark. Viability of larvae was monitored every 24 hours, for four days. To determine whether the larvae are alive or dead, they were gently touched with tweezers. If no movement was observed, larvae were considered to be dead [52]. Virulence was analysed by comparing the endpoint survival means (using one-way ANOVA with Bonferroni correction) and comparing the survival curves over time (by Kaplan-Meier estimate with log-rank test). The statistical analyses were performed using the SPSS Statistics software package and *survival* package implemented in R.

To compare doubling times, 28 *C*. *orthopsilosis* isolates were grown overnight in YPD broth in a shaking incubator at 30°C. Cultures were diluted to an A_600_ of 0.1 and incubated in a 96 well round bottom plate. A_600_ measurements were taken every 15 min over 48 h using a shaking plate reader (Biotek Synergy HT: Multi-Detection Microplate Reader), to generate growth curves. The doubling times of the isolates were calculated from three biological replicates, each with three technical replicates, using the software tool GATHODE [53].

### Sequencing and variant analysis

Library preparation and Illumina sequencing of 27 *C*. *orthopsilosis* isolates in two Illumina HiSeq 2500 lanes (150 bp paired-end) was carried out by the DNA Core Facility, University of Missouri, USA. Five isolates (Sample 427, Sample 831, Sample 422, Sample 282 and Sample 320) were sequenced on one lane, and the remaining samples were multiplexed in the second lane. Raw read numbers ranged from 40.59 to 69.22 million reads for the first five genomes and from 6.19 to 13.17 million reads for the remainder (Table 1). Raw reads were downloaded for previously published *C*. *orthopsilosis* genomes 90–125 [39] (Illumina GAIIX, 75 bp single end) and MCO456 [40] (Illumina HiSeq 2000, 100 bp paired-end). For each isolate, raw reads were trimmed using Skewer v0.1.117 [54] with parameters—quiet (without progress updates) -m pe (paired-end mode) -l 36 (minimum read length allowed after trimming) -q 15 (trim 3’ end of read until a quality of 15 or higher is reached) -Q 15 (lowest mean quality for a read allowed before trimming) and -t 4 (number of threads).

Reads were mapped to the reference genome (90–125) using the mem algorithm from bwa (with -t 8 threads and default options), generating BAM files for each isolate. AddOrReplaceReadGroups from Picard Tools v1.82 from the Broad Institute [55] was used to add read groups to BAM files in order to pass requirements of Genome Analysis Toolkit (GATK) for BAM files. BAM files were indexed using Samtools [56].

The GATK HaploTypeCaller [57] (with -nct 42 threads and default options) was used to obtain a high quality set of single nucleotide variants (SNVs). Identified SNVs from all samples were merged using GATK CombineVariants. For the SNP analysis, insertions and deletions were removed using a custom script. Each genome was divided into 1 kb windows and assigned to haplotype A, haplotype B or haplotype A/B depending on the number of homozygous and heterozygous SNPs compared to the reference 90–125, using a custom R script. Regions were defined as homozygous A if they had <10 homozygous SNPs and <10 heterozygous SNPs, homozygous B if >10 homozygous SNPs and <10 heterozygous SNPs, heterozygous A/B if <10 homozygous SNPs and >10 heterozygous SNPs, and undefined (>10 homozygous SNPs, >10 heterozygous SNPs). Undefined regions ranged from 0.93% (Sample 424) to 4.81% (Sample 434), which correlates with the overall heterozygosity levels (Pearson correlation coefficient of 0.86). Undefined regions probably arise because the 1 kb windows used sometimes span the start or the end of a LOH event. These 1 kb regions then correspond partly to a heterozygous region and partly to a homozygous region.

To infer haplotype B, all 1 kb regions in Sample 424 defined as either haplotype B or haplotype A/B were extracted. For heterozygous SNPs, the base that differed from 90–125 was used. In total, SNPs from 10.71 Mb were extracted. For each extracted SNP the base in the 90-125-reference genome was substituted with the base from haplotype B. GATK HaplotypeCaller was used to call SNPs for all isolates against the inferred parent B. SNPs in 1 kb regions were binned as described above.

Mitochondrial genomes were identified as contigs in genome assemblies made using Platanus v1.2.1 [58] and annotated by reference to Kosa et al [49]. Linear mtDNAs assembled as contigs ending in telomeric (*tel*) repeats (Fig 6). The circular mtDNAs present in two strains assembled as contigs containing junctions between the left and right subtelomeric (*sub*) regions and lacking telomeric repeats. All 28 mitochondrial genomes that we sequenced have been submitted to the EMBL database (accession numbers LT594353-LT594380).

### Copy number variation analysis

BAM files generated in the variant analysis step were used to characterize depth of coverage using the DepthOfCoverage tool in GATK with default parameters. Expected genome-wide coverage was calculated using the total number of mapped reads multiplied by the read length and divided by the size of the reference genome (90–125). Log_2_ ratios for copy number analysis for each isolate were calculated in 1 kb windows using the formula: log_2_ (observed coverage in 1 kb window / expected coverage) + log_2_ (total expected coverage / total observed coverage). Log ratios were then smoothed using the smooth.CNA() function of the DNAcopy package in Bioconductor (R package version 1.42.0). We used circular binary segmentation as implemented in the DNAcopy package to extract regions of equal copy number. All identified CNVs were manually verified.

### Phylogenetic analysis

The genomes were divided into 1 kb windows and, for each isolate, all SNPs (heterozygous and homozygous SNPs) in a specific 1 kb region were extracted if that region contained more than ten heterozygous SNPs in 20 or more of the analyzed isolates (excluding the two homozygous strains 90–125 and Sample 428). We identified 57530 SNPs from 1195 kb (9.43% of the genome). The SNPs were converted to a FASTA file and split into parental alleles (named A and B) using a custom script. In brief, if a base was identical to 90–125 it was assigned to A and an N was inserted for B; a homozygous SNP was assigned to B and an N was inserted for A; a heterozygous SNP was split into A (equal to 90–125) and B. If neither base of a heterozygous SNP matched 90–125 they were alphabetically ordered (A to T) and the first base was assigned to A and the second to B. RAxML v8.1.21 (raxmlHPC-PTHREADS) [45] was used to generate 20 maximum likelihood trees (options -m GTRCAT -p 12345) and 1000 bootstraps (-m GTRCAT -p 12345 -b 12345). Bipartitions were calculated by drawing all bootstraps onto the best maximum likelihood tree using RAxML (-m GTRCAT -p 12345 -fb). Trees were visualized using FigTree v1.4.2 (http://tree.bio.ed.ac.uk/software/figtree/)

### Identification of structural variation

To identify structural variations, including insertions, deletions and intra- and inter-chromosomal rearrangements, we utilized BreakDancer [59] with the BAM files generated in the variant analysis step. The script bam2cfg.pl from BreakDancer was used with 10000 random paired-end reads for each isolate to generate configuration files that list read length as well as lower, upper and mean insert size of the paired-end read fragments and its standard deviation. The insert size ranged from 412.01 to 442.29 bases, with a standard deviation between 95.09 and 109.56 bases. BreakDancer was then executed with default parameters and the results were analyzed manually.

### Whole genome sequencing and assembly using Pacific Biosciences RS II long reads

For PacBio sequencing of Sample 427, SMRT-bells generation, quality control and sequencing on two SMRT Cells using P6-C4 chemistry was outsourced to GATC Biotech Ltd., Constance, Germany. PacBio SMRT Portal version 2.3.0.140936.p2.144836 was used for quality assessment of reads, generation of subreads, genome assembly using the Hierarchical Genome Assembly Pipeline (HGAP3) algorithm and AHA (A Hybrid Approach) scaffolding. The SMRT portal was locally modified to allow execution of commands on a single 48 core Linux server with 256 GB of memory. A total of 300584 polymerase reads were generated from two SMRT Cells, with a total read base count of 1.09 billion and a N50 of 18093 bases. After filtering (with parameters minimum subread length of 500, minimum polymerase read quality of 0.8 and a minimum polymerase read length of 100), 79850 polymerase reads with a total read base count of 894.95 million and a N50 value of 19514 bases were used to extract 165541 subreads with a total read base count of 875.25 million and a N50 of 6992 bases.

The subreads were used as input for the RS_HGAP_Assembly.3 protocol in the SMRT Portal (with parameters minimum seed read length of 6000, number of seed read chunks of six, alignment candidates per chunk of 10, total alignment candidates of 24 and minimum coverage for correction of six). The draft assembly contained 263 polished contigs with a N50 of 292.25 kb, a length of 17.14 Mb and a mean coverage of 46.62x. Scaffolding with the RS_AHA_Scaffolding.1 protocol using five iterations resulted in 241 scaffolds, a N50 of 322.40 kb, a length of 17.16 Mb and 22 gaps with a total length of 19.04 kb. The 34 longest scaffolds (ranging from 988.53 kb to 91.93 kb) had a total sum of 12.65 Mb, compared to a total sum of 12.66 Mb for the Co_90–125 assembly. The mitochondrial genome was represented in a single 33.78 kb contig.

## Supporting Information

S1 File Genome assembly and analysis. (DOCX)

S1 Fig Assembly error in *C*. *orthopsilosis* 90–125. Circos plot of two contigs from the PacBio assembly of Sample 427 mapped against 90–125. Regions of unitig_270 and unitig_292 from Sample 427 map to chromosome 2 and 6 of 90–125. The error resulted from the presence of two highly similar genes on chromosomes 2 and 6, with only 3% sequence difference, that were collapsed to one in the 90–125 assembly [39].
(PDF)

S2 Fig Most *C*. *orthopsilosis* isolates are hybrids. The distribution of homozygous SNPs in 1 kb regions relative to isolate 90–125 is shown for all isolates. The SNP distribution is bimodal, with some regions almost identical to 90–125, and some regions that differ by >3%. These most likely represent the A and B haplotypes. When all regions are taken into account, the A and B haplotypes differ by 5.1%. Because only homozygous SNP are shown there is little data available from the highly heterozygous isolates, Sample 282, Sample 320, Sample 424, Sample 498, and Sample 748.
(PDF)

S3 Fig There is no evidence of a third parental species. 84.6% (or 10.7 Mb) of the parental B haplotype was inferred from Sample 424 (see Methods). The blue background represents regions where the parental B genome could not be inferred. Each isolate was then compared to both 90–125 (haplotype A) and the inferred haplotype B. The red lines show the number of homozygous SNPs (Y-axis) in 1 kb windows that are not present in either parent. Most SNPs were identified in regions where the B haplotype could not be inferred. There are some regions on chromosomes 7 and 8 (highlighted with black arrows) that differ from both 90–125 and the inferred B genome. These originate from LOH events in Sample 424 that are smaller than 1 kb (100–400 bases), and so are an artifact of the method used to infer the B haplotype. Strains are ordered according to clades and each chromosome is shown on a different page. Chromosomal location is shown on the X-axis.
(PDF)

S4 Fig There is no evidence of recombination in Sample 427 relative to 90–125. 90–125 represents an ancestral A genome. Five heterozygous regions from the PacBio assembly of Sample 427, that assembled into separate “A” and “B” contigs, are shown. For each, red ticks show SNP sites where the upper contig (haplotype A) matches 90–125 but differs from the lower contig (haplotype B). Conversely, blue ticks show SNP sites where the lower contig (haplotype B) matches 90–125 but differs from the upper contig (haplotype A). This analysis shows that, at least in these regions, the A and B haplotypes, which were defined by matching or not matching to 90–125, correspond to the parental haplotypes of the hybrid without recombination. Two regions of unitig_29 are shown. For more detail, see S1 Table.
(PDF)

S5 Fig Summary of genotypes and SNPs at the MTL (mating-type like) locus. Magenta and green boxes denote MTLα and MTLa idiomorphs, respectively. Blue lines and box outlines indicate DNA that has been derived from parent A. Brown lines and box outlines indicate DNA that has been derived from parent B. Vertical tick marks represent the presence of SNPs relative to the 90–125 reference genome sequence (SNP sites and gene locations are schematic and are not drawn to scale). In all clades, the parent-of-origin of the retained MTL idiomorphs (as shown by the blue or brown outlines) correlates with the parent-of-origin of the DNA flanking MTL on each side, except for a short region to the right of MTL in Clade 2. In Clade 4, Sample 498 represents a straightforward MTL heterozygote where the MTLα idiomorph came from Parent A and the MTLa idiomorph came from Parent B. Sample 424 may have been derived from a strain like 498 by LOH. In Clade 2, Sample 425 represents an MTL heterozygote where the MTLα idiomorph came from Parent B and the MTLa idiomorph came from Parent A (note that this is the converse of the hybridization in Clade 4). In this heterozygote, a small region of LOH to the right of MTL has made genes *CORT0E05760* and part of *CORT0E5770* homozygous for the A-haplotype. Samples 426 and 504 may been derived from a 425-like strain by LOH through the entire MTL region, with the strains retaining opposite haplotypes. Sample 427 may have been derived from a 425-like strain by LOH extending leftwards from the *CORT0E05760* region through the MTL locus. The homozygous isolates 90–125 and Sample 428 are related to, but not part of, Clade 4 and Clade 1 respectively.
(PDF)

S6 Fig Doubling time of *C*. *orthopsilosis* isolates and survival in larvae of *Galleria mellonella*. A. Doubling times were calculated using GATHODE [53] from growth curves of three biological replicates with three technical replicates growing in YPD broth at 30°C shaking for 48 h. The graph shows the average and standard deviation. Each isolate is colored with respect to its clade. The two homozygous isolates are shown in purple. B. As described in Fig 7, larvae of *G*. *mellonella* were inoculated with 5 x 10^5^ cells of each *C*. *orthopsilosis* strain. The results of two independent experiments were combined to calculate survival rates (i.e. 40 larvae per strain). Percent of surviving larvae over time in each clade was plotted in a Kaplan-Meier survival curve. Comparison of the curves with log rank test showed a significant difference between Clade 1 and 2 (*p* = 9.36 x 10^−4^), Clade 1 and Clade 3 (*p* = 5.74 x 10^−3^), Clade 1 and Clade 4 (*p* = 9.5 x 10^−4^), Clade 2 and Clade 3 (*p* = 3.33 x 10^−16^) and Clade 2 and 4 (*p* = 1.11 x 10^−16^). C. Kaplan-Meier curve comparing the virulence of the homozygous strains 90–125 and sample 428. Survival rates were calculated by combining two experiments. Curves were compared with log rank test, (*p* = 1.35 x 10^−10^).
(PDF)

S7 Fig Identification of aneuploidy and polyploidy in *C*. *orthopsilosis* Isolates. A. The distribution of frequencies of the B allele from single nucleotide variants are shown for all isolates (between 0.2 and 0.8). Diploid genomes have a peak at 0.5. Sample 282, highlighted with a red box, has three peaks, at 0.25, 0.5 and 0.75, indicating a tetraploid genome. The two homozygous isolates (90–125 and Sample 428) have an unusual distribution because they contain very few variants. Frequency distribution plots for each strain were generated using R [60]. B. Tetraploidy of Sample 282. For each SNP site on each chromosome, the fraction of mapped reads with SNPs is shown (left-hand axis), where 100% indicates homozygous SNPs different to 90–125; 50% denotes a 2:2 ratio of biallelic SNPs; and 25% and 75% denote 1:3 ratio or 3:1 ratio of biallelic SNPs. Histograms of coverage are shown on the right hand side of each panel. Horizontal lines indicate 25% and 75%. The coverage of all mapped reads is shown on the bottom. Red colored bars represent homozygous A or B regions (5 kb or longer, average fraction of mapped reads with SNPs is <15% or >85%), blue colored bars indicate heterozygous diploid A/B regions (fraction of mapped reads with SNPs is between 40% and 60%) and green bars show heterozygous tetraploid regions (fraction of mapped reads with SNPs is between 60–85% or between 15–40%). C. Trisomy of chromosome 7 in Sample 437. Log ratios of expected vs. actual coverage, with 1 kb sliding windows shown as a red line. Chromosomes 1 to 8 are ordered from left to right in alternating colors. For clarity only log ratios from -2 to 2 are shown.
(PDF)

S8 Fig Copy number variation in *C*. *orthopsilosis*. One region of chromosome 1 is amplified six times and an adjacent region is amplified three times in Sample 185. The plots show the read coverage (Y-axis) for Sample 185 and 90–125. The region on the left (0.5X coverage) is heterozygous (A/B) in Sample 185. There are 3 copies of a gene of unknown function at the left-hand end of the 6X region in *C*. *parapsilosis* and in most isolates of *C*. *orthopsilosis*; the amplification results in 18 copies in the haploid genome of Sample 185 (one copy, *CORT0A07110*, indicated with *, is incorrectly annotated in 90–125). At least some of the copies on the 6X region are amplified in inverse orientation, indicated by the presence of reads with hairpin loops at the left hand edge. The 3X region is surrounded by an inverted repeat (highlighted in red boxes). Some of the ORFs in this region were not annotated in 90–125; the most likely *C*. *parapsilosis* orthologs are shown.
(PDF)

S9 Fig Identification of inteins in the threonyl-tRNA synthetase gene *THS1* in *C*. *orthopsilosis* isolates. The wiggle plots show the read coverage (Y-axis) around the *THS1* gene (drawn to scale) for 28 *C*. *orthopsilosis* isolates. Clades are colored as green (Clade 1), red (Clade 2), orange (Clade 3), and blue (Clade 4). The mini intein contains only the self-splicing blocks A, B, F and G (blue boxes). The full-length intein also includes the homing domains C, D, E and H (indicated by a thin blue line in *THS1*). Each diploid isolate contains two *THS1* alleles, which may have either full-length (F) or mini (M) inteins, or are heterozygous.
(PDF)

S1 Table Comparison of phased regions of the PacBio Assembly of Sample 427. (DOCX)

S2 Table Characteristics of mitochondrial genome in *C*. *orthopsilosis* isolates. (DOCX)

S3 Table List of Copy Number Variations in *C*. *orthopsilosis* isolates. (DOCX)
