# Supplementary material for: Multiple Origins of the Pathogenic Yeast Candida orthopsilosis by Separate Hybridizations between Two Parental Species
Source: PLoS Genet. 2016 Nov 2;12(11):e1006404. doi: 10.1371/journal.pgen.1006404 (PMC5091853; doi:10.1371/journal.pgen.1006404)
Supplement: S1 File — (DOCX) [file pgen.1006404.s001.docx]

**Supporting File S1.**

**Genome assemblies**

Read data was mapped to the *C. orthopsilosis* 90-125 reference genome using BWA, as described in Materials and Methods. In addition, *de novo* assemblies were generated using Platanus [1]. These assemblies were not used in the analyses reported except for the mitochondrial genomes. The PacBio assembly of sample 427 was generated as described in the main Methods section. The assembly contained 241 scaffolds, an N50 of 322 kb, and 22 gaps, with a total length of 17.2 Mb. The 34 longest scaffolds (ranging from 989 kb to 92 kb) totalled 12.65 Mb, compared to an estimate of 12.66 Mb for the 90-125 reference assembly. The high level of heterozygosity in strain 427 resulted in a very fragmented assembly, primarily with breakpoints at the borders of LOH events, with many reading frame errors. We attempted to use the Illumina data from sample 427 to correct the errors in the PacBio sequence. However, the PacBio coverage (46.6X), was not sufficient to confidently assemble both haplotypes across the entire genome.

**Copy number variations**

We used Single Nucleotide Polymorphisms (SNP) allele counts within each isolate to estimate ploidy [2]. The distribution of B-allele frequencies are shown for all isolates in S7A Fig. Most isolates have a single peak around 0.5 indicative of a diploid genome [3]. The two homozygous isolates (90-125 and Sample 428) are unusual because they contain very few variants. Sample 282, highlighted with a red box in S7A Fig, has three peaks, at 0.25, 0.5 and 0.75, indicating a tetraploid genome. Although the SNP data is consistent with most of the other isolates being diploid, we cannot exclude the possibility that some of them might be recently-formed tetraploids with 0.5 (2:2) allele ratios. A closer analysis of Sample 282 confirms that for many SNPs, one allele is present in 25% of the reads, and the second allele in 75% (Fig. S7B; see histograms on the right-hand side of each panel). The 25%/75% ratio was observed in sections of most of the 8 chromosomes, except in regions with loss of heterozygosity, suggesting that the entire genome of this isolate is tetraploid. In addition, comparing the read density of each isolate against the 90-125 reference genome [4] allowed us to identify trisomy of chromosome 7 in one isolate (Sample 437) (Fig. S7C).

Several small copy number variations were also identified (S3 Table). Fifteen apparent CNVs are due to assembly errors in 90-125 where repetitive regions were collapsed into a single consensus, which were also described by Pryszcz et al [5]. Overall, we identified an additional 55 CNVs > 1 kb across all isolates relative to the reference genome 90-125 (S3 Table). In Sample 185 a region of 21 kb on chromosome 1 is amplified six times, and an adjacent region of 12 kb is present in three copies (S8 Fig, S3 Table). A predicted P-type ATPase sodium pump (*CORT0H02780*), which is found near the telomere of chromosome 8 in 90-125, is amplified in three strains from Clade 1 (Sample 1799, Sample 434 and Sample 423). This amplification was described in MCO456 by Pryszcz et al [5], suggesting that it is specific to Clade 1. There is also a large amplification (up to 40 copies) around genes CORT0B05830/CORT0B05840 in several isolates (S3 Table). These genes are members of the RTA family, and likely encode flippases involved in sphingolipid long chain base release. In *C. albicans*, this family has been associated with resistance to azoles and other drugs [6].

**Identification of intein polymorphisms in *C. orthopsilosis***

Inteins are regions of proteins that are excised by an autocatalytic process after translation [7]. Prandini et al [8] showed that the length of the intein in the Vma1 vacuolar ATPase protein could be used to distinguish species within the *C. parapsilosis* species complex. They also reported that an intein in *THS1* (threonyl-tRNA aminoacyl synthetase, ThrRS) occurs in two versions in *C. orthopsilosis* isolates, mini- and full-length. We characterized both inteins (*THS1* (*CORT0B02480*) and *VMA1* (*CORT0D07070*)) in the 27 sequenced isolates. No differences in the *VMA1* intein were identified. Some isolates contain only the full-length *THS1* intein, some only the mini intein, and some are heterozygous for both (S9 Fig). The intein pattern correlates with the clade designation described in Fig 2.

References

1. Kajitani R, Toshimoto K, Noguchi H, Toyoda A, Ogura Y, Okuno M, et al. Efficient de novo assembly of highly heterozygous genomes from whole-genome shotgun short reads. Genome Res. 2014;24:1384-95.

2. Rupp O, Brinkrolf K, Buerth C, Kunigo M, Schneider J, Jaenicke S, et al. The structure of the *Cyberlindnera jadinii* genome and its relation to *Candida utilis* analyzed by the occurrence of single nucleotide polymorphisms. J Biotechnol. 2015;211:20-30.

3. Yoshida K, Schuenemann VJ, Cano LM, Pais M, Mishra B, Sharma R, et al. The rise and fall of the *Phytophthora infestans* lineage that triggered the Irish potato famine. Elife. 2013;2:e00731.

4. Riccombeni A, Vidanes G, Proux-Wéra E, Wolfe KH, Butler G. Sequence and analysis of the genome of the pathogenic yeast *Candida orthopsilosis*. PLoS ONE. 2012;7:e35750.

5. Pryszcz LP, Németh T, Gácser A, Gabaldón T. Genome comparison of *Candida orthopsilosis* clinical strains reveals the existence of hybrids between two distinct subspecies. Genome Biol Evol. 2014;6:1069-78.

6. Jia XM, Wang Y, Jia Y, Gao PH, Xu YG, Wang L, et al. RTA2 is involved in calcineurin-mediated azole resistance and sphingoid long-chain base release in Candida albicans. Cell Mol Life Sci. 2009;66:122-34.

7. Chong S, Shao Y, Paulus H, Benner J, Perler FB, Xu M-Q. Protein splicing involving the *Saccharomyces cerevisiae* VMA intein. J Biol Chem. 1996;271:22159-68.

8. Prandini THR, Theodoro RC, Bruder-Nascimento ACMO, Scheel CM, Bagagli E. Analysis of inteins in the *Candida parapsilosis* complex for simple and accurate species identification. J Clin Microbiol. 2013;51:2830-6.
