# Supplementary figures and images for: Multiple Origins of the Pathogenic Yeast Candida orthopsilosis by Separate Hybridizations between Two Parental Species
Source: PLoS Genet. 2016 Nov 2;12(11):e1006404. doi: 10.1371/journal.pgen.1006404 (PMC5091853; doi:10.1371/journal.pgen.1006404)

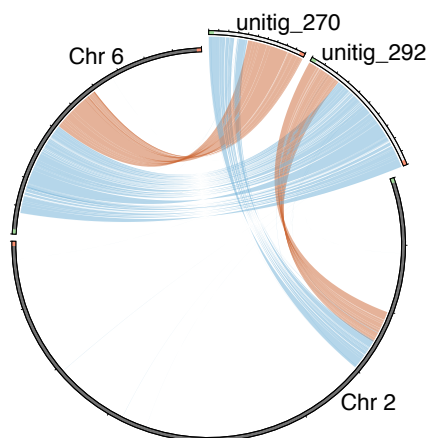

**S1 Fig.** Assembly error in *C. orthopsilosis* 90-125.

Supplement: S1 Fig — Circos plot of two contigs from the PacBio assembly of Sample 427 mapped against 90–125. Regions of unitig_270 and unitig_292 from Sample 427 map to chromosome 2 and 6 of 90–125. The error resulted from the presence of two highly similar genes on chromosomes 2 and 6, with only 3% sequence difference, that were collapsed to one in the 90–125 assembly [39]. (PDF) [file pgen.1006404.s002.pdf]

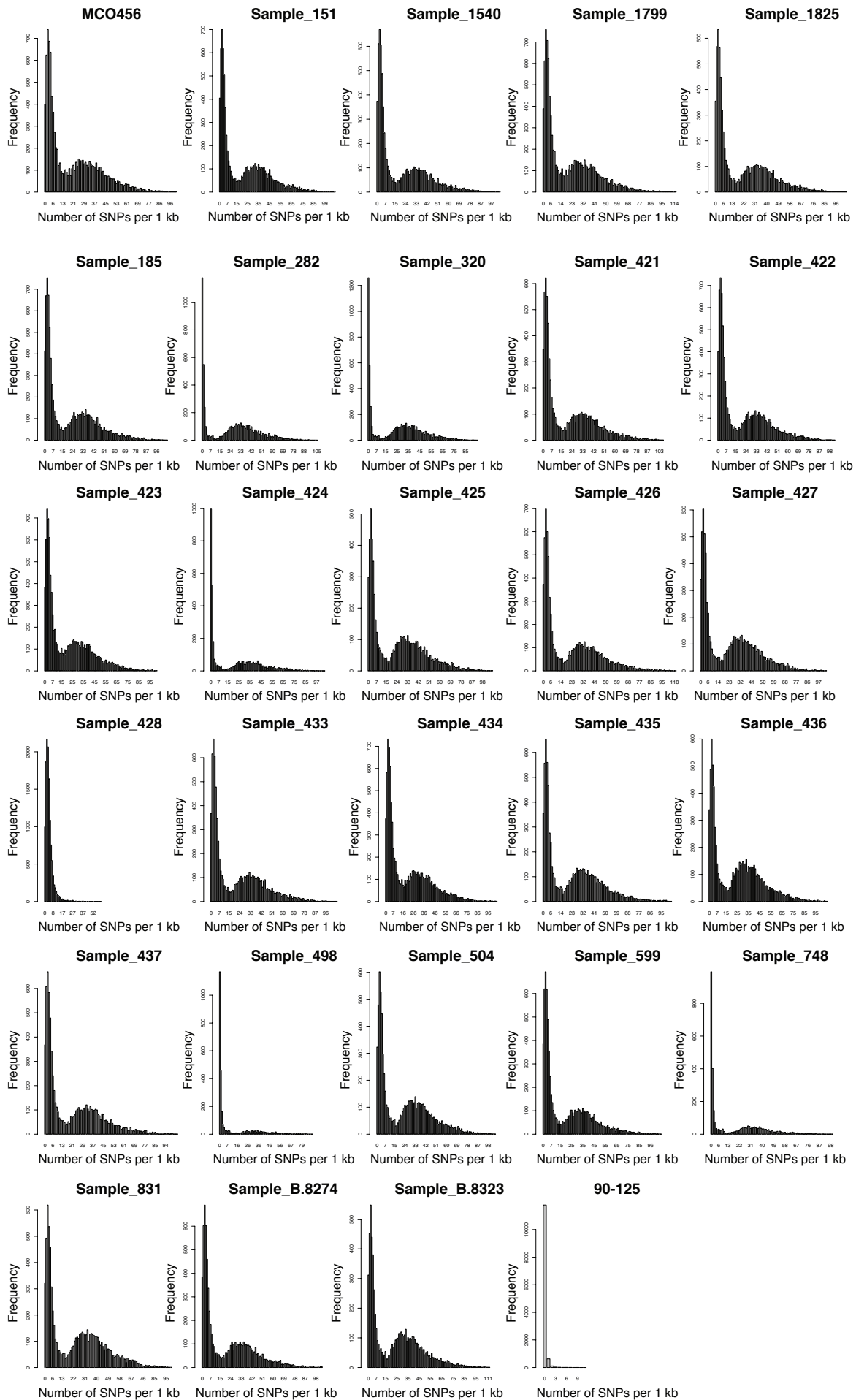

**S2 Fig.** Most *C. orthopsilosis* isolates are hybrids.

Supplement: S2 Fig — The distribution of homozygous SNPs in 1 kb regions relative to isolate 90–125 is shown for all isolates. The SNP distribution is bimodal, with some regions almost identical to 90–125, and some regions that differ by >3%. These most likely represent the A and B haplotypes. When all regions are taken into account, the A and B haplotypes differ by 5.1%. Because only homozygous SNP are shown there is little data available from the highly heterozygous isolates, Sample 282, Sample 320, Sample 424, Sample 498, and Sample 748. (PDF) [file pgen.1006404.s003.pdf]

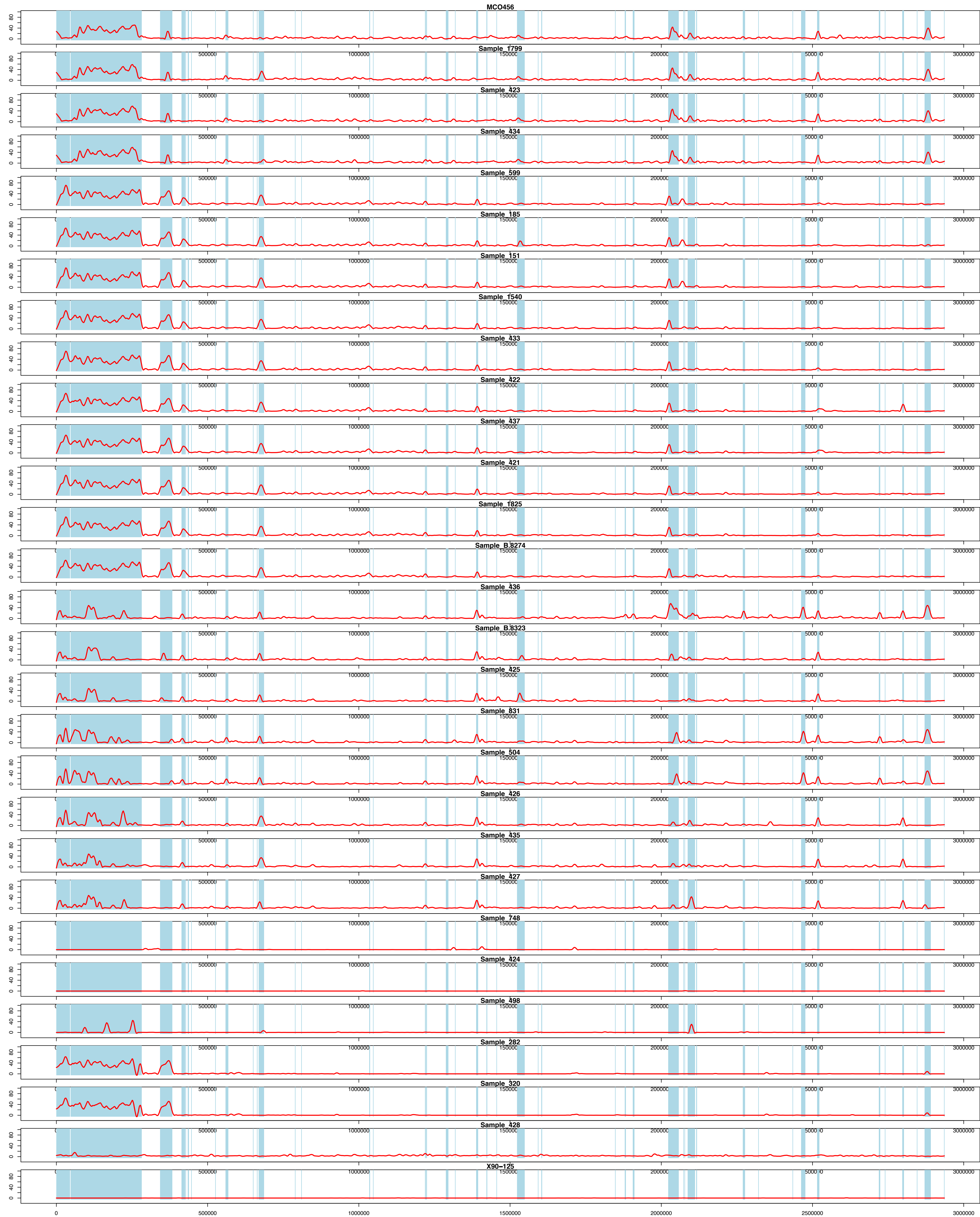

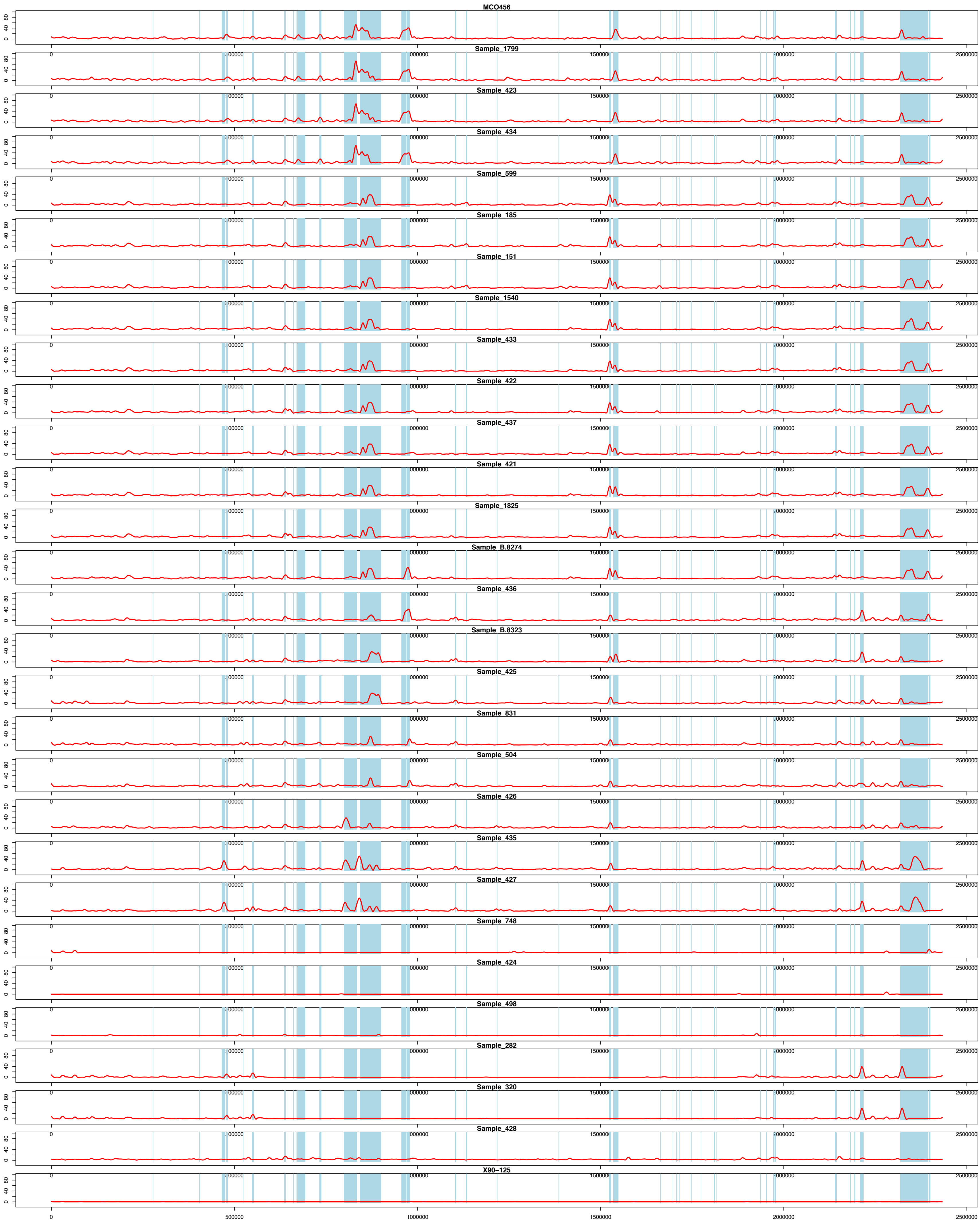

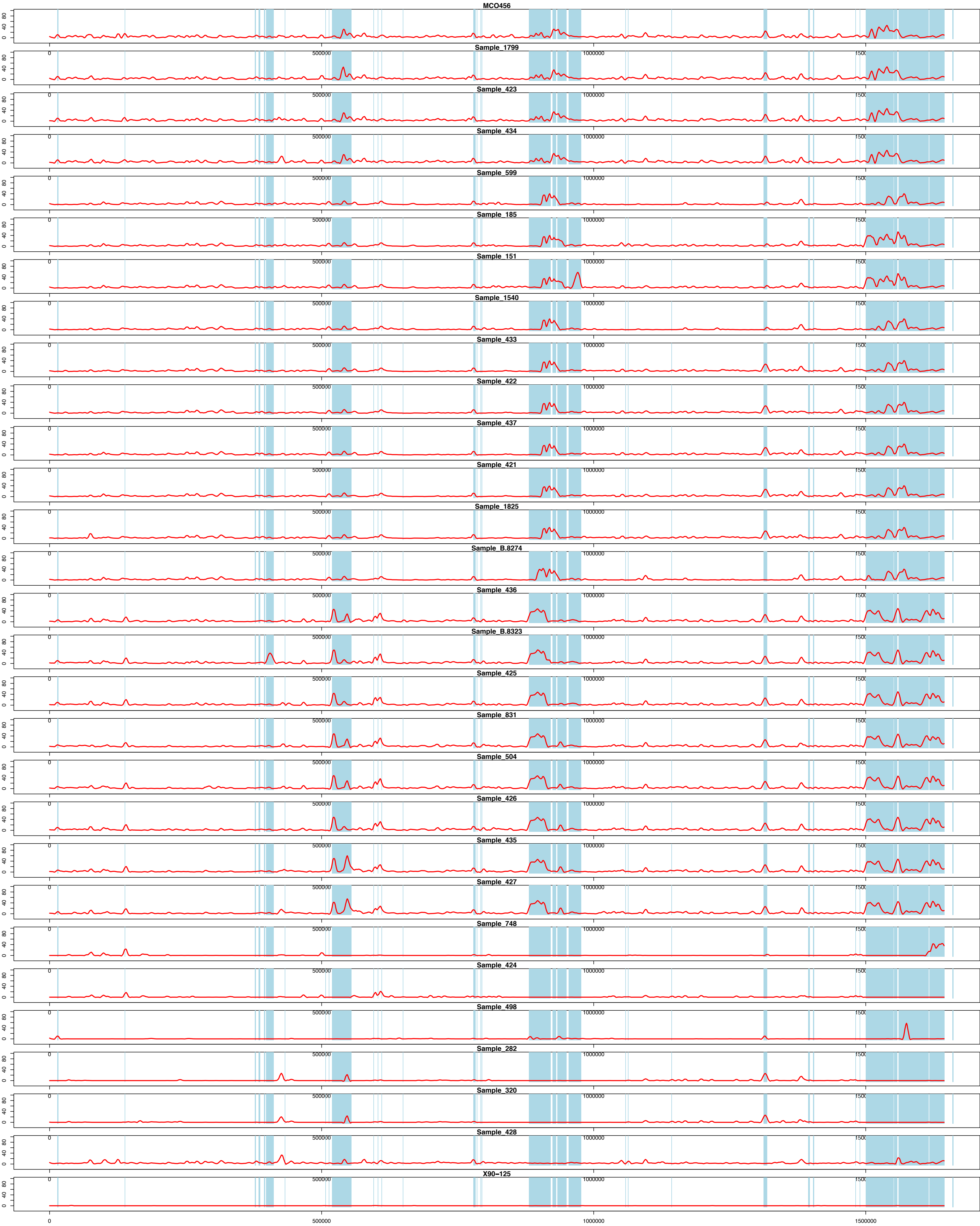

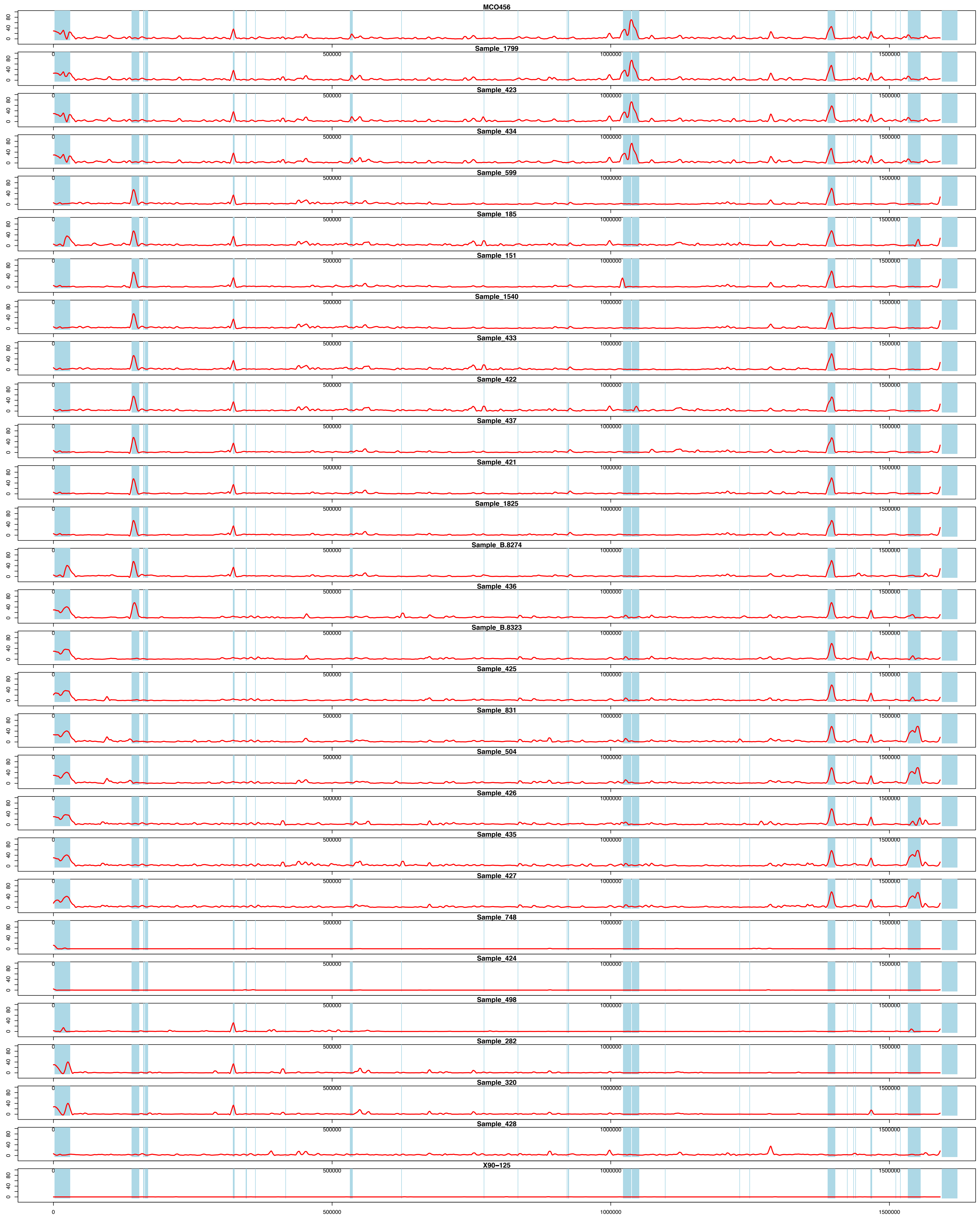

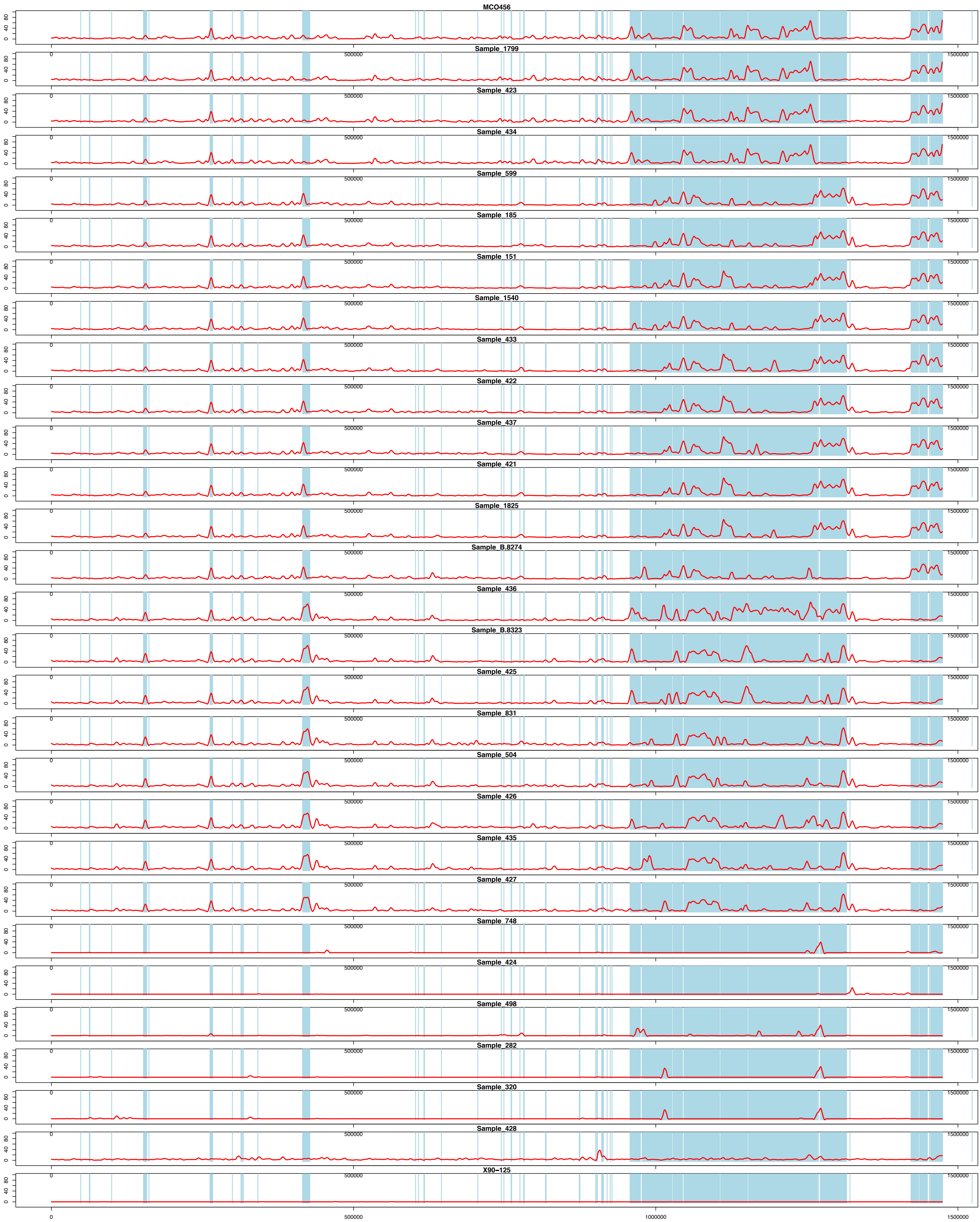

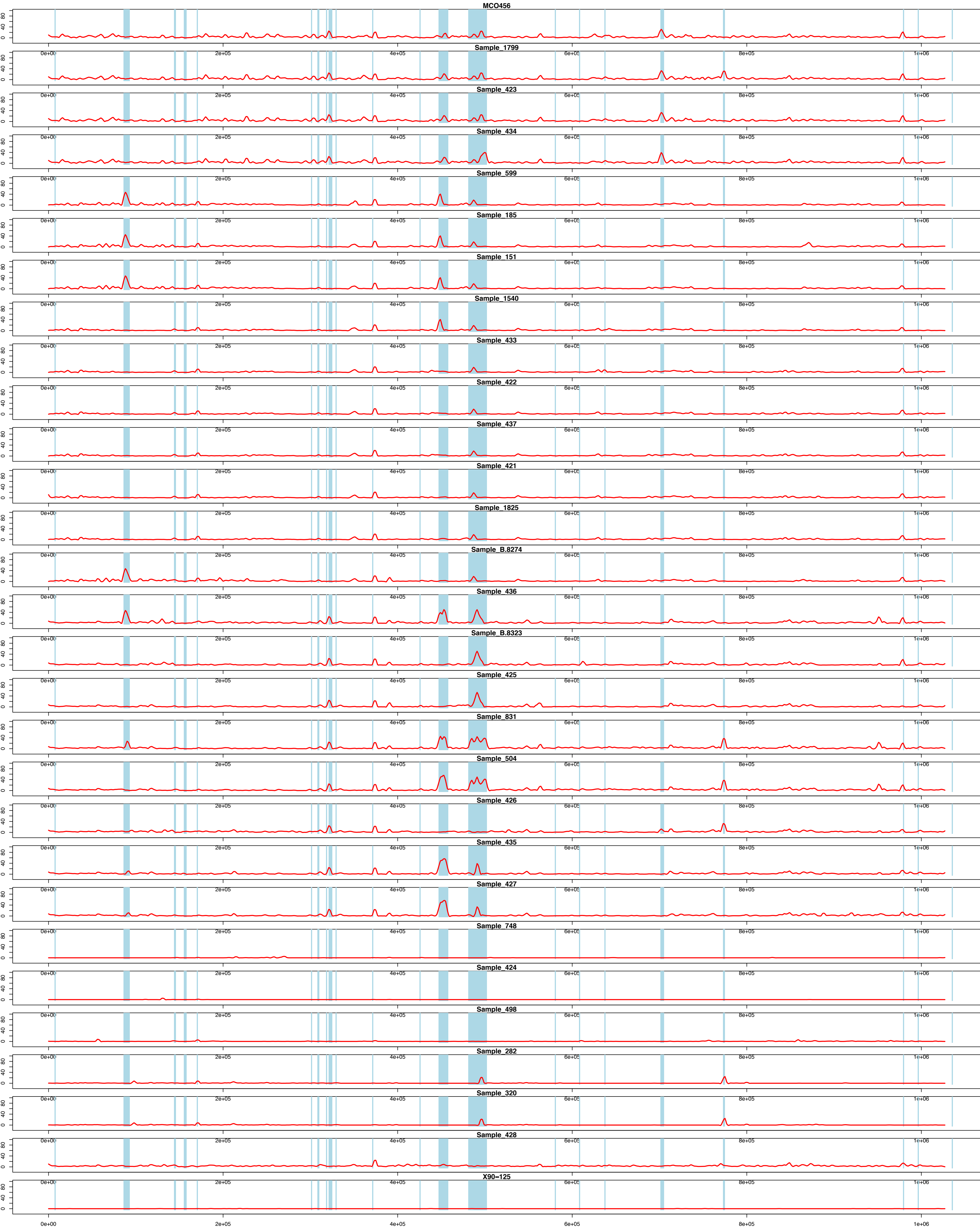

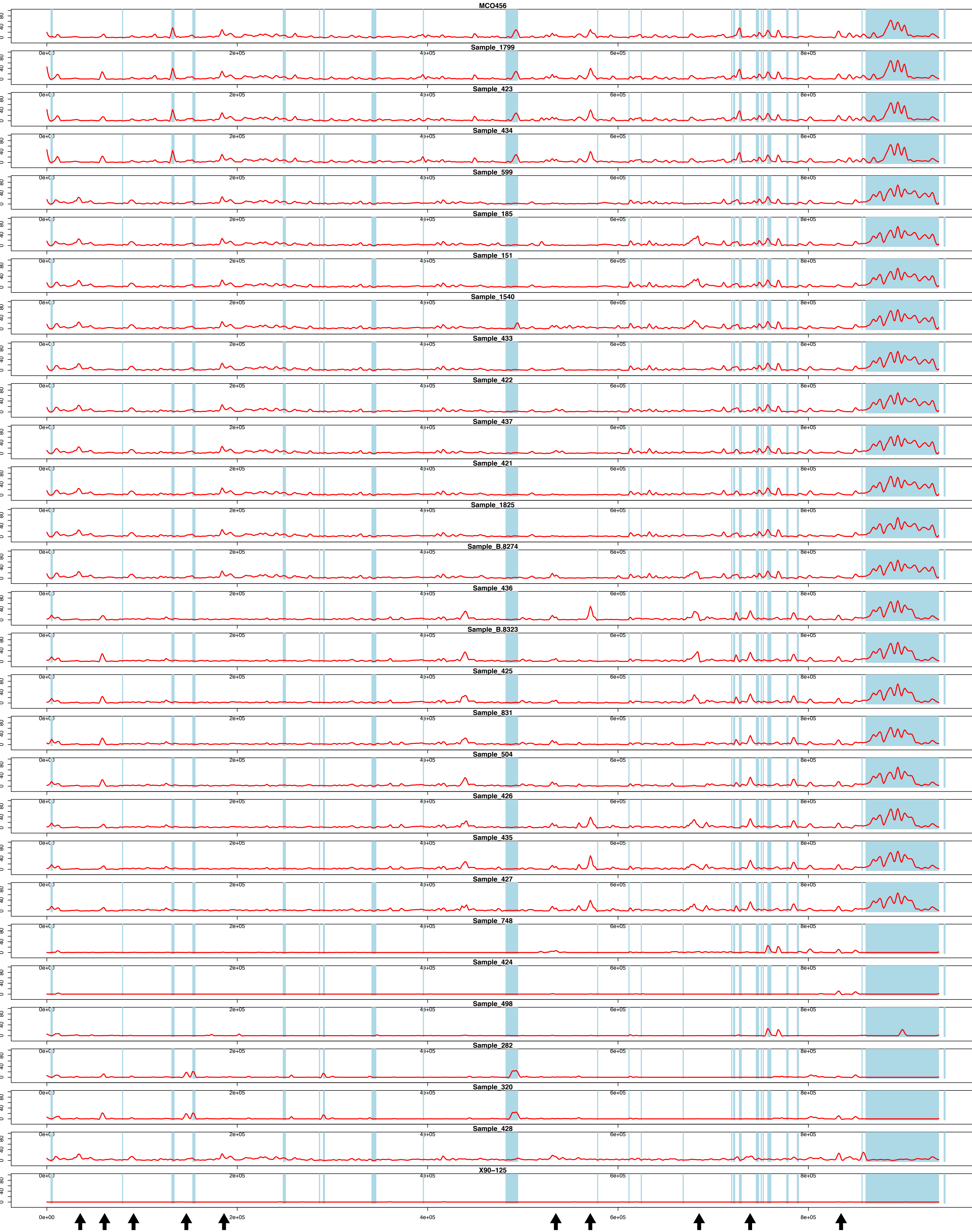

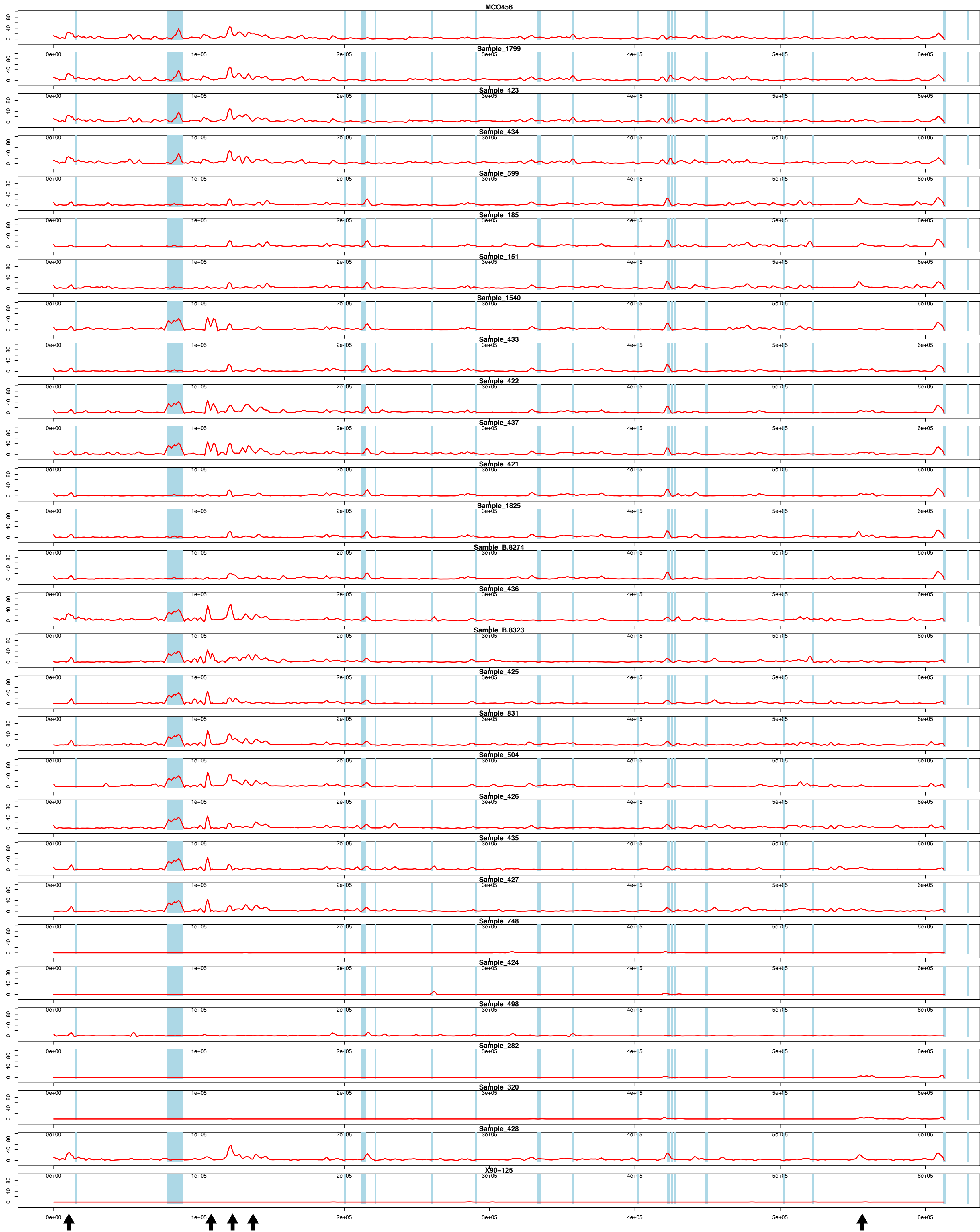

**S3 Fig.** There is no evidence of a third parental species.

Supplement: S3 Fig — 84.6% (or 10.7 Mb) of the parental B haplotype was inferred from Sample 424 (see Methods). The blue background represents regions where the parental B genome could not be inferred. Each isolate was then compared to both 90–125 (haplotype A) and the inferred haplotype B. The red lines show the number of homozygous SNPs (Y-axis) in 1 kb windows that are not present in either parent. Most SNPs were identified in regions where the B haplotype could not be inferred. There are some regions on chromosomes 7 and 8 (highlighted with black arrows) that differ from both 90–125 and the inferred B genome. These originate from LOH events in Sample 424 that are smaller than 1 kb (100–400 bases), and so are an artifact of the method used to infer the B haplotype. Strains are ordered according to clades and each chromosome is shown on a different page. Chromosomal location is shown on the X-axis. (PDF) [file pgen.1006404.s004.pdf]

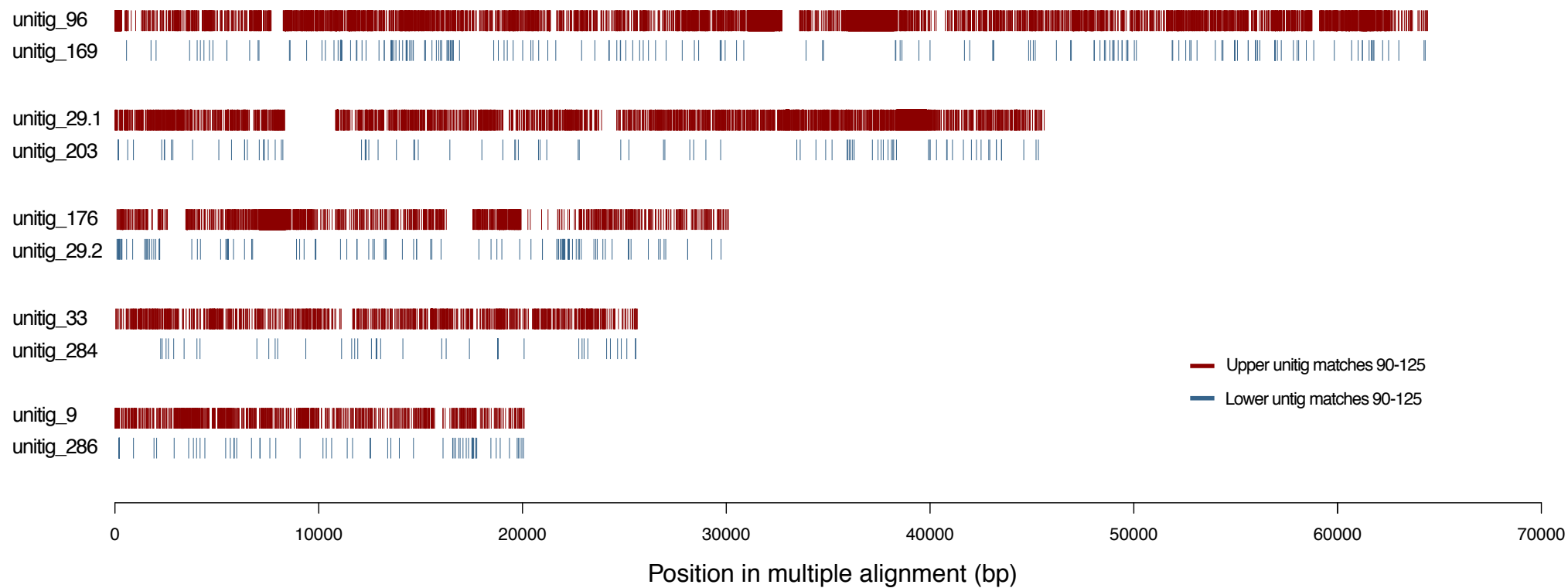

**S4 Fig.** There is no evidence of recombination in Sample 427 relative to 90-125

Supplement: S4 Fig — 90–125 represents an ancestral A genome. Five heterozygous regions from the PacBio assembly of Sample 427, that assembled into separate “A” and “B” contigs, are shown. For each, red ticks show SNP sites where the upper contig (haplotype A) matches 90–125 but differs from the lower contig (haplotype B). Conversely, blue ticks show SNP sites where the lower contig (haplotype B) matches 90–125 but differs from the upper contig (haplotype A). This analysis shows that, at least in these regions, the A and B haplotypes, which were defined by matching or not matching to 90–125, correspond to the parental haplotypes of the hybrid without recombination. Two regions of unitig_29 are shown. For more detail, see S1 Table. (PDF) [file pgen.1006404.s005.pdf]

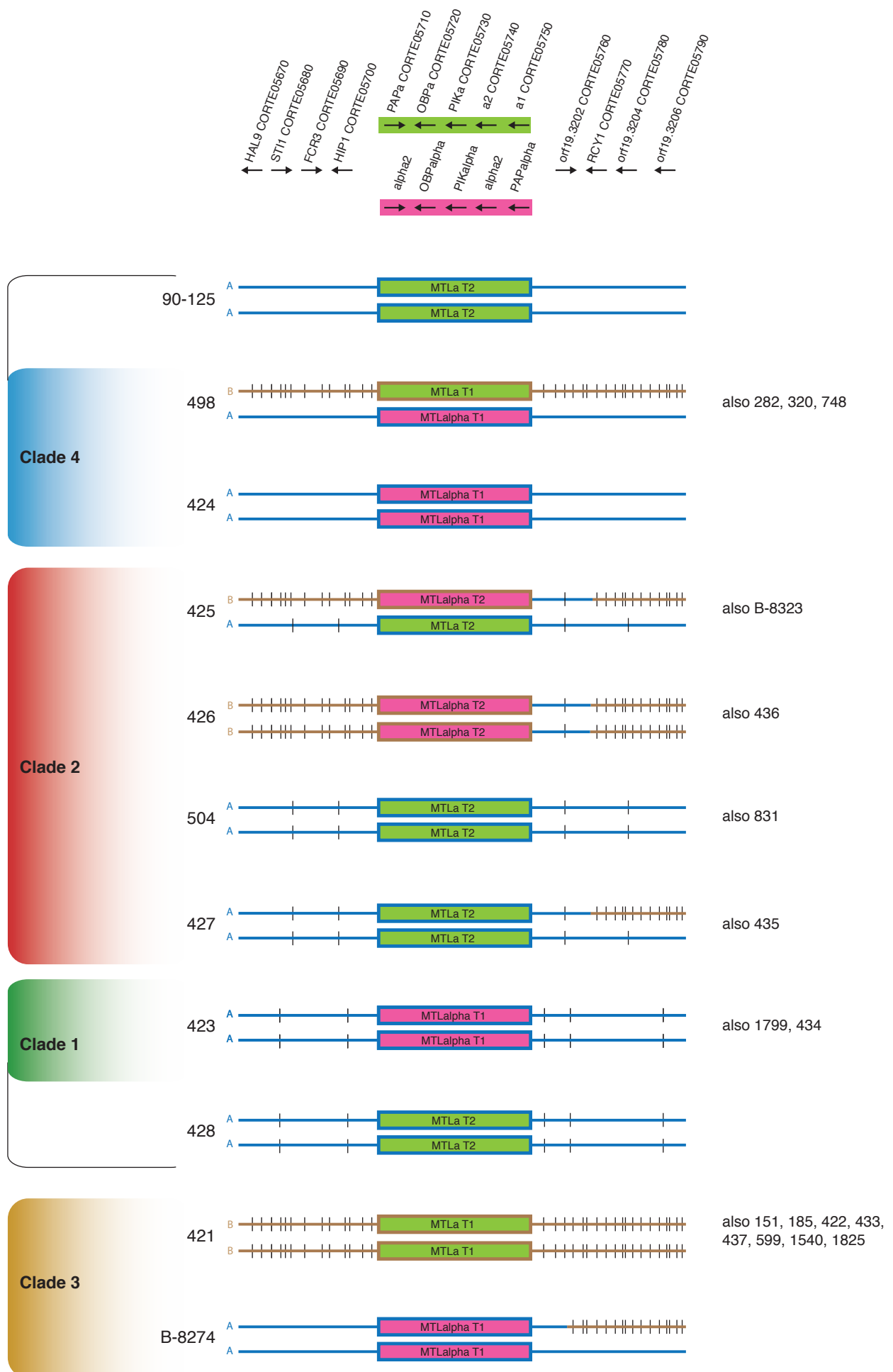

**S5 Fig.** Summary of genotypes and SNPs at the MTL (mating-type like) locus.

Supplement: S5 Fig — Magenta and green boxes denote MTLα and MTLa idiomorphs, respectively. Blue lines and box outlines indicate DNA that has been derived from parent A. Brown lines and box outlines indicate DNA that has been derived from parent B. Vertical tick marks represent the presence of SNPs relative to the 90–125 reference genome sequence (SNP sites and gene locations are schematic and are not drawn to scale). In all clades, the parent-of-origin of the retained MTL idiomorphs (as shown by the blue or brown outlines) correlates with the parent-of-origin of the DNA flanking MTL on each side, except for a short region to the right of MTL in Clade 2. In Clade 4, Sample 498 represents a straightforward MTL heterozygote where the MTLα idiomorph came from Parent A and the MTLa idiomorph came from Parent B. Sample 424 may have been derived from a strain like 498 by LOH. In Clade 2, Sample 425 represents an MTL heterozygote where the MTLα idiomorph came from Parent B and the MTLa idiomorph came from Parent A (note that this is the converse of the hybridization in Clade 4). In this heterozygote, a small region of LOH to the right of MTL has made genes CORT0E05760 and part of CORT0E5770 homozygous for the A-haplotype. Samples 426 and 504 may been derived from a 425-like strain by LOH through the entire MTL region, with the strains retaining opposite haplotypes. Sample 427 may have been derived from a 425-like strain by LOH extending leftwards from the CORT0E05760 region through the MTL locus. The homozygous isolates 90–125 and Sample 428 are related to, but not part of, Clade 4 and Clade 1 respectively. (PDF) [file pgen.1006404.s006.pdf]
