# Supplementary material for: Multiple Origins of the Pathogenic Yeast Candida orthopsilosis by Separate Hybridizations between Two Parental Species
Source: PLoS Genet. 2016 Nov 2;12(11):e1006404. doi: 10.1371/journal.pgen.1006404 (PMC5091853; doi:10.1371/journal.pgen.1006404)
Supplement: S6 Fig — A. Doubling times were calculated using GATHODE [53] from growth curves of three biological replicates with three technical replicates growing in YPD broth at 30°C shaking for 48 h. The graph shows the average and standard deviation. Each isolate is colored with respect to its clade. The two homozygous isolates are shown in purple. B. As described in Fig 7, larvae of G. mellonella were inoculated with 5 x 10^5^ cells of each C. orthopsilosis strain. The results of two independent experiments were combined to calculate survival rates (i.e. 40 larvae per strain). Percent of surviving larvae over time in each clade was plotted in a Kaplan-Meier survival curve. Comparison of the curves with log rank test showed a significant difference between Clade 1 and 2 (p = 9.36 x 10^−4^), Clade 1 and Clade 3 (p = 5.74 x 10^−3^), Clade 1 and Clade 4 (p = 9.5 x 10^−4^), Clade 2 and Clade 3 (p = 3.33 x 10^−16^) and Clade 2 and 4 (p = 1.11 x 10^−16^). C. Kaplan-Meier curve comparing the virulence of the homozygous strains 90–125 and sample 428. Survival rates were calculated by combining two experiments. Curves were compared with log rank test, (p = 1.35 x 10^−10^). (PDF) [file pgen.1006404.s007.pdf]

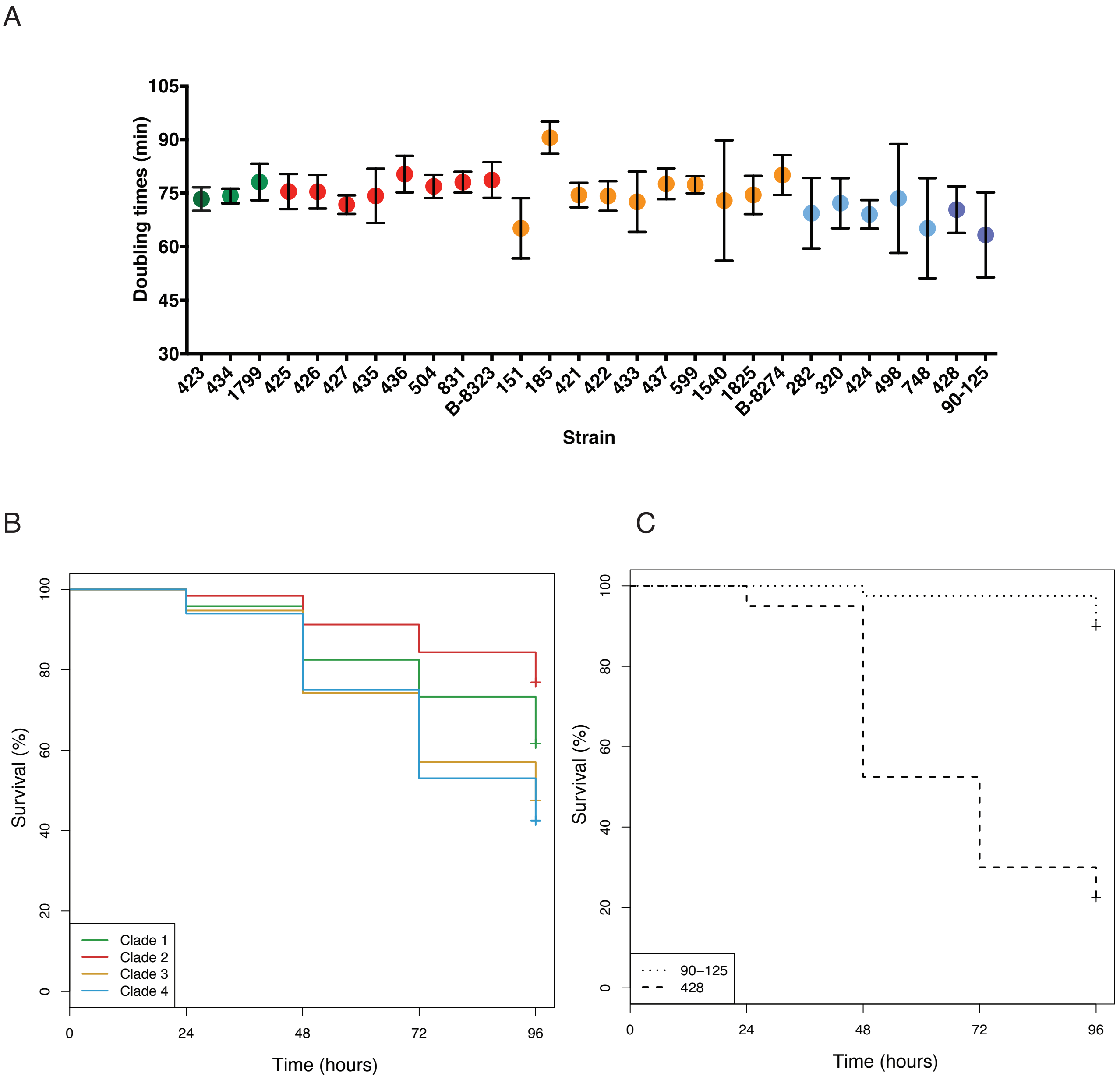

S6 Fig. Doubling time of *C. orthopsilosis* isolates and survival in larvae of *Galleria mellonella*.
