# Supplementary material for: Multiple Origins of the Pathogenic Yeast Candida orthopsilosis by Separate Hybridizations between Two Parental Species
Source: PLoS Genet. 2016 Nov 2;12(11):e1006404. doi: 10.1371/journal.pgen.1006404 (PMC5091853; doi:10.1371/journal.pgen.1006404)
Supplement: S7 Fig — A. The distribution of frequencies of the B allele from single nucleotide variants are shown for all isolates (between 0.2 and 0.8). Diploid genomes have a peak at 0.5. Sample 282, highlighted with a red box, has three peaks, at 0.25, 0.5 and 0.75, indicating a tetraploid genome. The two homozygous isolates (90–125 and Sample 428) have an unusual distribution because they contain very few variants. Frequency distribution plots for each strain were generated using R [60]. B. Tetraploidy of Sample 282. For each SNP site on each chromosome, the fraction of mapped reads with SNPs is shown (left-hand axis), where 100% indicates homozygous SNPs different to 90–125; 50% denotes a 2:2 ratio of biallelic SNPs; and 25% and 75% denote 1:3 ratio or 3:1 ratio of biallelic SNPs. Histograms of coverage are shown on the right hand side of each panel. Horizontal lines indicate 25% and 75%. The coverage of all mapped reads is shown on the bottom. Red colored bars represent homozygous A or B regions (5 kb or longer, average fraction of mapped reads with SNPs is <15% or >85%), blue colored bars indicate heterozygous diploid A/B regions (fraction of mapped reads with SNPs is between 40% and 60%) and green bars show heterozygous tetraploid regions (fraction of mapped reads with SNPs is between 60–85% or between 15–40%). C. Trisomy of chromosome 7 in Sample 437. Log ratios of expected vs. actual coverage, with 1 kb sliding windows shown as a red line. Chromosomes 1 to 8 are ordered from left to right in alternating colors. For clarity only log ratios from -2 to 2 are shown. (PDF) [file pgen.1006404.s008.pdf]

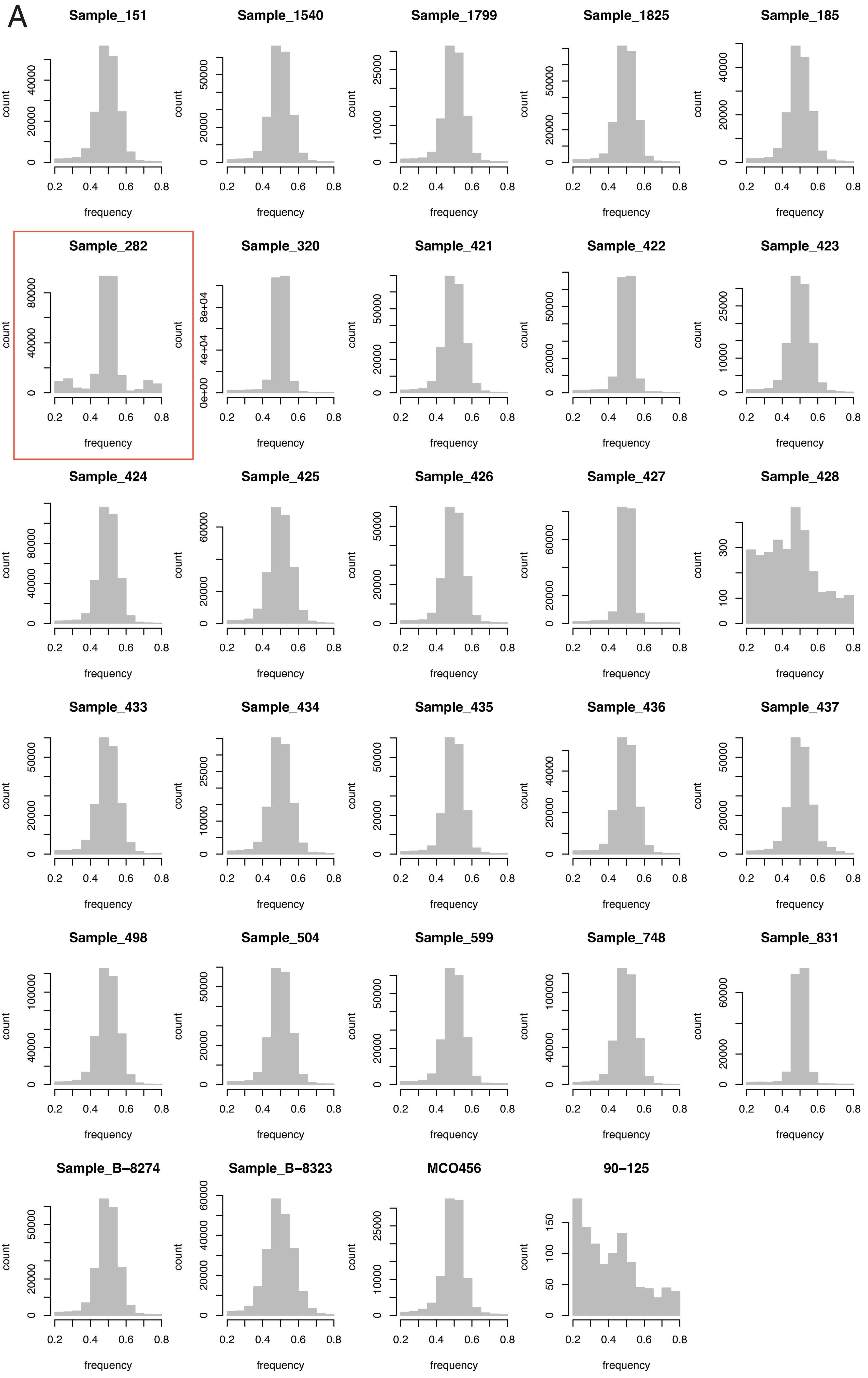

A
Sample_151
Sample_1540
Sample_1799
Sample_1825
Sample_185
Sample_282
Sample_320
Sample_421
Sample_422
Sample_423
Sample_424
Sample_425
Sample_426
Sample_427
Sample_428
Sample_433
Sample_434
Sample_435
Sample_436
Sample_437
Sample_498
Sample_504
Sample_599
Sample_748
Sample_831
Sample_B–8274
Sample_B–8323
MCO456
90–125
count
frequency

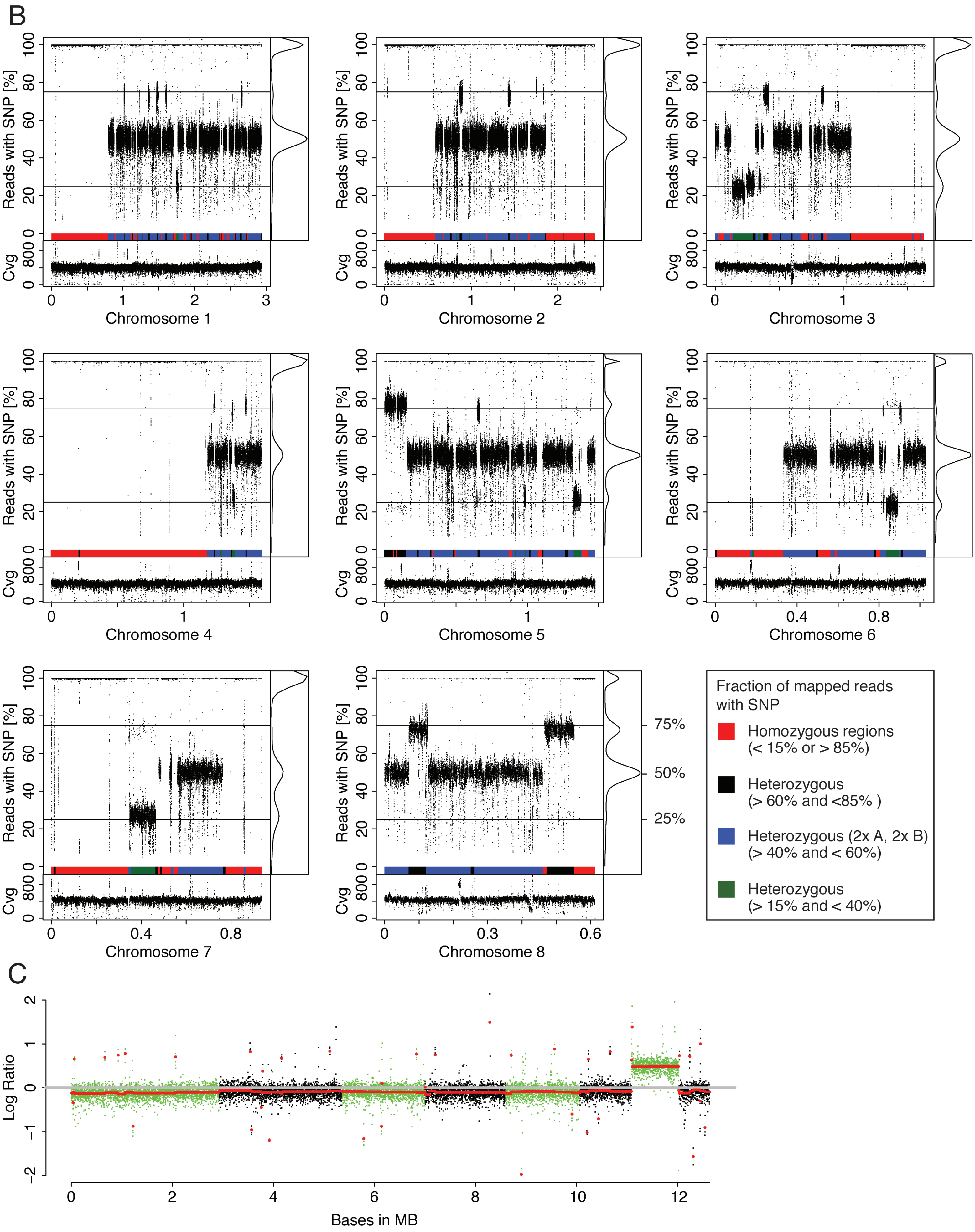
B
Reads with SNP [%]
Cvg
Chromosome 1
Chromosome 2
Chromosome 3
Chromosome 4
Chromosome 5
Chromosome 6
Chromosome 7
Chromosome 8
75%
50%
25%
Fraction of mapped reads with SNP
Homozygous regions (< 15% or > 85%)
Heterozygous (> 60% and <85%)
Heterozygous (2x A, 2x B) (> 40% and < 60%)
Heterozygous (> 15% and < 40%)
C
Log Ratio
Bases in MB

S7 Fig. Identification of aneuploidy and polyploidy in *C. orthopsilosis* isolates.

A. The distribution of frequencies of the B allele from single nucleotide variants are shown for all isolates (between 0.2 and 0.8). Diploid genomes have a peak at 0.5. Sample 282, highlighted with a red box, has three peaks, at 0.25, 0.5 and 0.75, indicating a tetraploid genome. The two homozygous isolates (90-125 and Sample 428) have an unusual distribution because they contain very few variants. Frequency distribution plots for each strain were generated using R [60].

B. Tetraploidy of Sample 282. For each SNP site on each chromosome, the fraction of mapped reads with SNPs is shown (left-hand axis), where 100% indicates homozygous SNPs different to 90-125; 50% denotes a 2:2 ratio of biallelic SNPs; and 25% and 75% denote 1:3 ratio or 3:1 ratio of biallelic SNPs. Histograms of coverage are shown on the right hand side of each panel. Horizontal lines indicate 25% and 75%. The coverage of all mapped reads is shown on the bottom. Red colored bars represent homozygous A or B regions (5 kb or longer, average fraction of mapped reads with SNPs is <15% or >85%), blue colored bars indicate heterozygous diploid A/B regions (fraction of mapped reads with SNPs is between 40% and 60%) and green bars show heterozygous tetraploid regions (fraction of mapped reads with SNPs is between 60-85% or between 15-40%).

C. Trisomy of chromosome seven in Sample 437. Log ratios of expected vs. actual coverage, with 1 kb sliding windows shown as a red line. Chromosomes 1 to 8 are ordered from left to right in alternating colors. For clarity only log ratios from -2 to 2 are shown.
