# Supplementary material for: Multiple Origins of the Pathogenic Yeast Candida orthopsilosis by Separate Hybridizations between Two Parental Species
Source: PLoS Genet. 2016 Nov 2;12(11):e1006404. doi: 10.1371/journal.pgen.1006404 (PMC5091853; doi:10.1371/journal.pgen.1006404)
Supplement: S8 Fig — One region of chromosome 1 is amplified six times and an adjacent region is amplified three times in Sample 185. The plots show the read coverage (Y-axis) for Sample 185 and 90–125. The region on the left (0.5X coverage) is heterozygous (A/B) in Sample 185. There are 3 copies of a gene of unknown function at the left-hand end of the 6X region in C. parapsilosis and in most isolates of C. orthopsilosis; the amplification results in 18 copies in the haploid genome of Sample 185 (one copy, CORT0A07110, indicated with *, is incorrectly annotated in 90–125). At least some of the copies on the 6X region are amplified in inverse orientation, indicated by the presence of reads with hairpin loops at the left hand edge. The 3X region is surrounded by an inverted repeat (highlighted in red boxes). Some of the ORFs in this region were not annotated in 90–125; the most likely C. parapsilosis orthologs are shown. (PDF) [file pgen.1006404.s009.pdf]

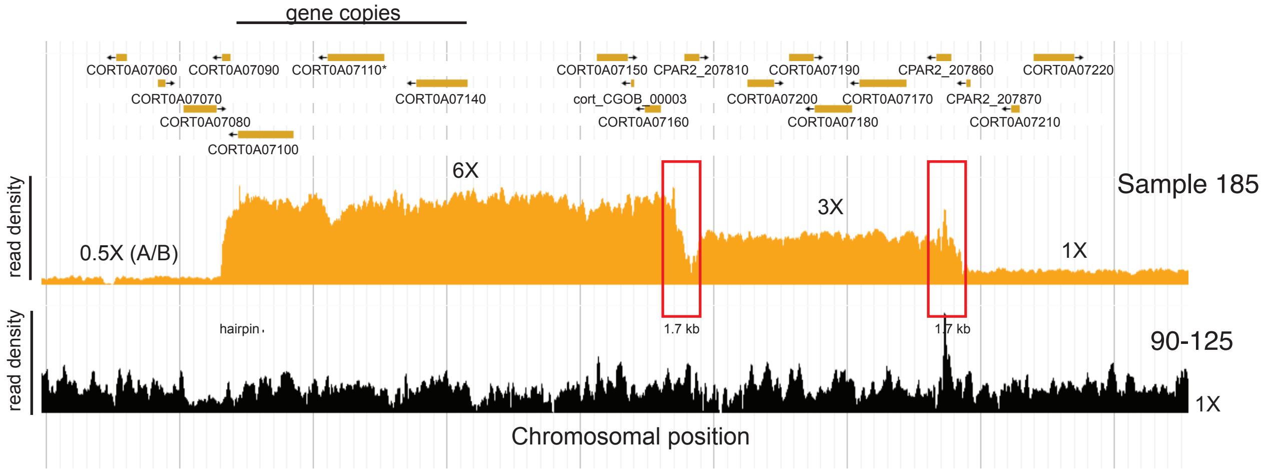

S8 Fig. Copy number variation in *C. orthopsilosis*

One region of chromosome 1 is amplified six times and an adjacent region is amplified three times in Sample 185. The plots show the read coverage (Y-axis) for Sample 185 and 90-185. The region on the left (0.5X coverage) is heterozygous (A/B) in Sample 185. There are 3 copies of a gene of unknown function at the left-hand end of the 6X region in *C. parapsilosis* and in most isolates of *C. orthopsilosis*; the amplification results in 18 copies in the haploid genome of Sample 185 (one copy, CORT0A07110, indicated with *, is incorrectly annotated in 90-125). At least some of the copies on the 6X region are amplified in inverse orientation, indicated by the presence of reads with hairpin loops at the left hand edge. The 3X region is surrounded by an inverted repeat (highlighted in red boxes). Some of the ORFs in this region were not annotated in 90-125; the most likely *C. parapsilosis* orthologs are shown.
