# Supplementary material for: Multiple Origins of the Pathogenic Yeast Candida orthopsilosis by Separate Hybridizations between Two Parental Species
Source: PLoS Genet. 2016 Nov 2;12(11):e1006404. doi: 10.1371/journal.pgen.1006404 (PMC5091853; doi:10.1371/journal.pgen.1006404)
Supplement: S9 Fig — The wiggle plots show the read coverage (Y-axis) around the THS1 gene (drawn to scale) for 28 C. orthopsilosis isolates. Clades are colored as green (Clade 1), red (Clade 2), orange (Clade 3), and blue (Clade 4). The mini intein contains only the self-splicing blocks A, B, F and G (blue boxes). The full-length intein also includes the homing domains C, D, E and H (indicated by a thin blue line in THS1). Each diploid isolate contains two THS1 alleles, which may have either full-length (F) or mini (M) inteins, or are heterozygous. (PDF) [file pgen.1006404.s010.pdf]

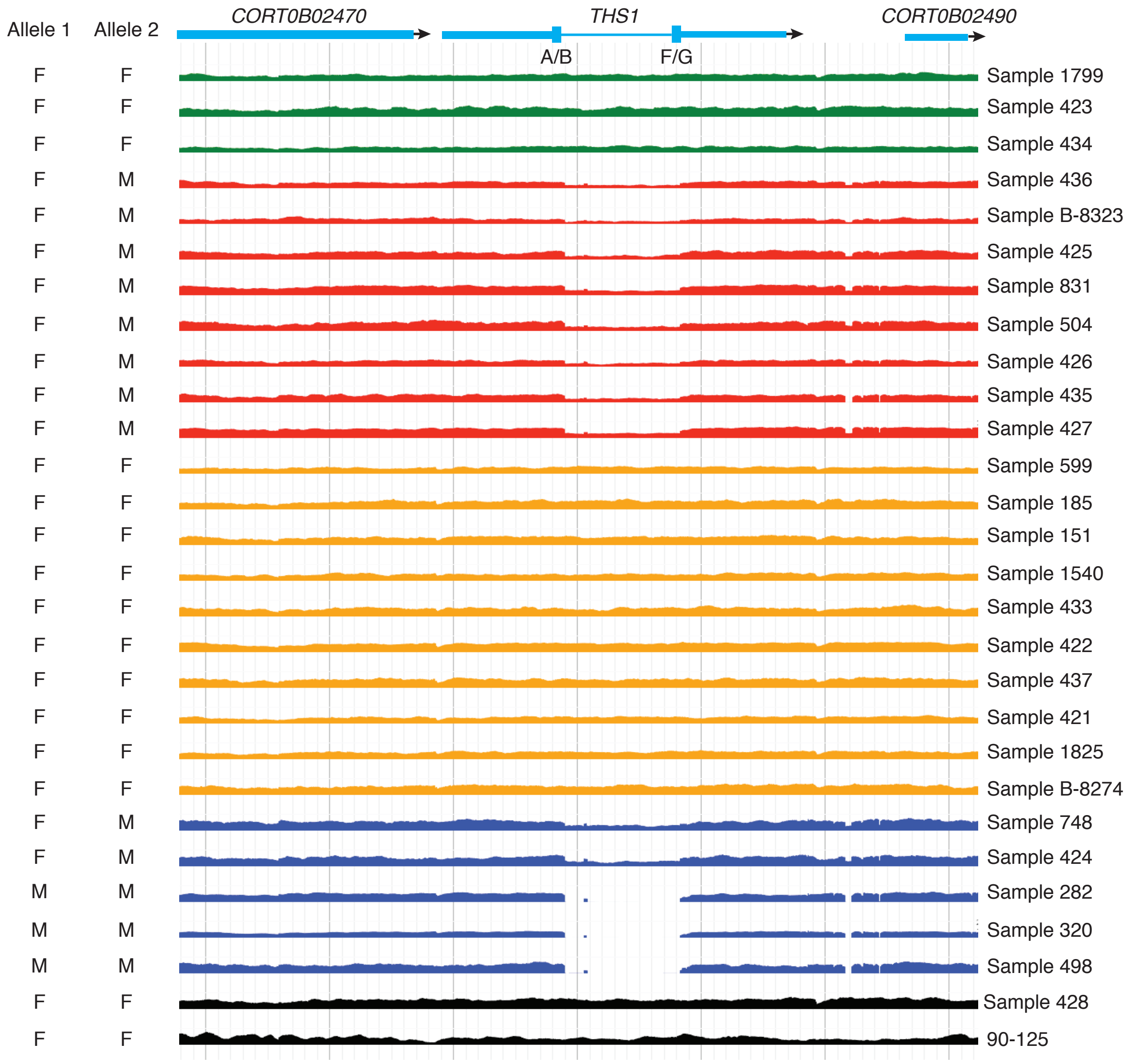

S9 Fig. Identification of inteins in the threonyl-tRNA synthetase gene *THS1* in *C. orthopsilosis* isolate

The wiggle plots show the read coverage (Y-axis) around the *THS1* gene (drawn to scale) for 28 *C. orthopsilosis isolates*. Clades are colored as green (Clade 1), red (Clade 2), orange (Clade 3), and blue (Clade 4). The mini intein contains only the self-splicing blocks A, B, F and G (blue boxes). The full-length intein also includes the homing domains C, D, E and H (indicated by a thin blue line in THS1). Each diploid isolate contains two *THS1* alleles, which may have either full-length (F) or mini (M) inteins, or are heterozygous.
