# Supplementary material for: Multiple Origins of the Pathogenic Yeast Candida orthopsilosis by Separate Hybridizations between Two Parental Species
Source: PLoS Genet. 2016 Nov 2;12(11):e1006404. doi: 10.1371/journal.pgen.1006404 (PMC5091853; doi:10.1371/journal.pgen.1006404)
Supplement: S1 Table — (DOCX) [file pgen.1006404.s011.docx]

**Table S1.** Regions used for phasing of the PacBio genome assembly (Sample 427).

|  |  |  |  | Number of bases | | | |
| --- | --- | --- | --- | --- | --- | --- | --- |
| Type | Contig/Chr | Coordinates | Aln. length [bp] | Iden-tical | A eq Ref | B eq Ref | Ambi-guous |
| A | unitig_96 | 49000-109500 | 64448 | 56142 | 7825 | 244 | 237 |
| Ref | chr1 | 244855-305462 |  |  |  |  |  |
| B | unitig_169 | 1786-305462 |  |  |  |  |  |
| A | unitig_29 | 228000-272000 | 45618 | 40921 | 4145 | 105 | 447 |
| Ref | chr1 | 1465350-1509343 |  |  |  |  |  |
| B | unitig_203 | 1435-46639 |  |  |  |  |  |
| A | unitig_176 | 28904-236 | 30129 | 25469 | 2659 | 128 | 1873 |
| Ref | chr1 | 1297209-1324211 |  |  |  |  |  |
| B | unitig_29 | 45380-75200 |  |  |  |  |  |
| A | unitig_33 | 3698-29173 | 25638 | 24368 | 1112 | 49 | 109 |
| Ref | chr4 | 967601-994138 |  |  |  |  |  |
| B | unitig_284 | 24500-50000 |  |  |  |  |  |
| A | unitig_9 | 2093-22023 | 20124 | 18901 | 1051 | 69 | 103 |
| Ref | chr5 | 841059-860998 |  |  |  |  |  |
| B | unitig_286 | 104000-124000 |  |  |  |  |  |

Columns describe from left to right: origin of sequence (Sample 427 haplotype A (A), Sample 427 haplotype B (B) or strain 90-125 (Ref)); name of contig or chromosome; sequence coordinates (start-stop); Clustal Omega alignment length; number of bases identical between A, Ref and B; A and Ref identical but B different; B and Ref identical but A different; A and B different from Ref (ambiguous). The corresponding alignments are shown schematically in S4 Fig. Two heterozygous regions of untig 29, which are separated by a block of homozygosity, were analyzed.
