# Supplementary material for: Multiple Origins of the Pathogenic Yeast Candida orthopsilosis by Separate Hybridizations between Two Parental Species
Source: PLoS Genet. 2016 Nov 2;12(11):e1006404. doi: 10.1371/journal.pgen.1006404 (PMC5091853; doi:10.1371/journal.pgen.1006404)
Supplement: S2 Table — (DOCX) [file pgen.1006404.s012.docx]

|  |  | | | | | | |
| --- | --- | --- | --- | --- | --- | --- | --- |
| Sample | Form | ri2 intron^a^ | ri1 intron^a^ | 44 bp insert in *rrnL* | ai3 intron^a^ | Subterminal  repeat^b^ | Subterminal repeat and telomere length^b^ |
| 425 | Linear | + | + | + | + | TATT |  |
| 427 | Linear | + | + | + | + | TATT | 451 / 565 |
| 435 | Linear | + | + | + | + | TATT |  |
| 436 | Circular | + | + | + | + | TATT-derived |  |
| 504 | Linear | + | + | + | + | TATT |  |
| 831 | Linear | + | + | + | + | TATT |  |
| B-8323 | Linear | + | + | + | + | TATT |  |
| 426 | Linear | + | + | + | + | TATT |  |
| IFM48386 | Linear | + | + | + | + | TATT | 451 / 565 |
| 498 | Linear | no | no | no | no | TATTC |  |
| 90-125 | Circular | no | no | no | no | CTTA-derived |  |
| 282 | Linear | no | no | + | + | CTTA |  |
| 320 | Linear | no | no | + | + | CTTA |  |
| 424 | Linear | no | no | + | + | CTTA |  |
| 748 | Linear | no | no | + | + | CTTA |  |
| MCO471 | Linear | no | no | + | + | CTTA | 396 / 777 |
| 428 | Linear | no | no | no | no | CTTA |  |
| 423 | Linear | no | no | no | no | CTTA |  |
| 434 | Linear | no | no | no | no | CTTA |  |
| 1799 | Linear | no | no | no | no | CTTA |  |
| MCO456 | Circular | no | no | no | no | CTTA-derived |  |
| B-8274 | Linear | no | no | no | + | ACTT |  |
| 151 | Linear | no | no | no | + | ACTT |  |
| 185 | Linear | no | no | no | + | ACTT |  |
| 421 | Linear | no | no | no | + | ACTT |  |
| 422 | Linear | no | no | no | + | ACTT |  |
| 433 | Linear | no | no | no | + | ACTT |  |
| 437 | Linear | no | no | no | + | ACTT |  |
| 599 | Linear | no | no | no | + | ACTT |  |
| 1540 | Linear | no | no | no | + | ACTT |  |
| 1825 | Linear | no | no | no | + | ACTT |  |

**Table S2.** Characteristics of mitochondrial genomes in *C. orthopsilosis* isolates

^a^ Intron nomenclature follows Kosa et al (2006). Introns ri1 and ri2 in *rrnL*, and ai3 in *cox1*, are polymorphic for presence/absence in *C. orthopsilosis*. The ai4 and bi2 introns are invariably present in all strains (Fig. 6A).

^b^ Sequence of the first 4-5 bp of the subterminal repeat identifies two main subterminal sequence types: a 451 bp sequence beginning with TATT and a 396 bp sequence beginning with CTTA. The subterminal repeat of Sample 498 was not completely sequenced but is different from the TATT group and may represent a third type.
