# Supplementary material for: Multiple Origins of the Pathogenic Yeast Candida orthopsilosis by Separate Hybridizations between Two Parental Species
Source: PLoS Genet. 2016 Nov 2;12(11):e1006404. doi: 10.1371/journal.pgen.1006404 (PMC5091853; doi:10.1371/journal.pgen.1006404)
Supplement: S3 Table — (DOCX) [file pgen.1006404.s013.docx]

**Table S3.** List of Copy Number Variations in *C. orthopsilosis* isolates.

The numbers in the matrix are estimated copy numbers.

| **Chromosome co-ordinates** | **434** | **423** | **1799** | **425** | **B-8323** | **427** | **435** | **504** | **426** | **831** | **436** | **421** | **1825** | **B-8274** | **1540** | **433** | **599** | **437** | **151** | **422** | **185** | **748** | **424** | **282** | **320** | **498** | **428** | **90-125** | **Comment** |
| --- | --- | --- | --- | --- | --- | --- | --- | --- | --- | --- | --- | --- | --- | --- | --- | --- | --- | --- | --- | --- | --- | --- | --- | --- | --- | --- | --- | --- | --- |
| cort_c_1:1000..2000 | 2 | 2 | 2 | 2 | 2 | 2 | 2 | 2 | 2 | 2 | 2 | 2 | 2 | 2 | 2 | 2 | 2 | 2 | 2 | 2 | 2 | 3 | 2 | 2 | 2 | 3 | 2 | 2 | Telomeric region |
| cort_c_1:87000..88000 | 0 | 0 | 0 | 0 | 0 | 1 | 1 | 0 | 1 | 0 | 0 | 1 | 1 | 2 | 2 | 2 | 2 | 1 | 2 | 1 | 2 | 2 | 2 | 2 | 2 | 2 | 0 | 2 | Between gaps in reference genome |
| cort_c_1:128000..130000 | 0 | 2 | 2 | 2 | 2 | 1 | 1 | 2 | 1 | 2 | 1 | 2 | 2 | 2 | 2 | 2 | 2 | 2 | 2 | 2 | 2 | 2 | 2 | 2 | 2 | 2 | 2 | 2 | Between gaps in reference genome |
| cort_c_1:1290000..1295000 | 0 | 0 | 0 | 0 | 0 | 0 | 0 | 0 | 0 | 0 | 0 | 0 | 0 | 0 | 0 | 0 | 0 | 0 | 0 | 0 | 0 | 0 | 0 | 0 | 0 | 1 | 0 | 2 | Near gap in reference genome |
| cort_c_1:1406000..1408000 | 2 | 2 | 2 | 0 | 0 | 0 | 0 | 0 | 0 | 0 | 0 | 1 | 1 | 1 | 1 | 1 | 1 | 1 | 1 | 1 | 1 | 0 | 1 | 1 | 1 | 1 | 2 | 2 | Within CORT0A06350 |
| cort_c_1:1461000..1462000 | 4 | 4 | 4 | 2 | 2 | 4 | 4 | 4 | 4 | 4 | 4 | 4 | 4 | 4 | 4 | 4 | 4 | 4 | 4 | 4 | 4 | 4 | 4 | 4 | 4 | 4 | 2 | 2 | Copy number increase of gene CORT0A06610 |
| cort_c_1:1495000..1498000 | 2 | 2 | 2 | 1 | 2 | 2 | 2 | 2 | 2 | 2 | 2 | 2 | 2 | 2 | 2 | 2 | 2 | 2 | 2 | 2 | 2 | 2 | 2 | 2 | 2 | 2 | 2 | 2 | Missing CORT0A06770 in Sample_425 |
| cort_c_1:1571000..1592000 | 2 | 2 | 2 | 2 | 2 | 2 | 2 | 2 | 2 | 2 | 2 | 2 | 2 | 2 | 2 | 2 | 2 | 2 | 2 | 2 | 12 | 2 | 2 | 2 | 2 | 2 | 2 | 2 | Amplified iin Sample_185 |
| cort_c_1:1592000..1604000 | 2 | 2 | 2 | 2 | 2 | 2 | 2 | 2 | 2 | 2 | 2 | 2 | 2 | 2 | 2 | 2 | 2 | 2 | 2 | 2 | 6 | 2 | 2 | 2 | 2 | 2 | 2 | 2 | Amplified iin Sample_185 |
| cort_c_1:2069000..2077000 | 0 | 0 | 0 | 0 | 0 | 0 | 0 | 0 | 0 | 0 | 0 | 2 | 2 | 2 | 2 | 2 | 2 | 2 | 2 | 2 | 2 | 1 | 2 | 2 | 2 | 2 | 0 | 2 | Also includes gaps in the reference genome and is next to a collapsed copy CORT0A09370 |
| cort_c_1:2226000..2228000 | 2 | 2 | 2 | 2 | 2 | 2 | 2 | 2 | 2 | 2 | 2 | 2 | 2 | 2 | 2 | 2 | 2 | 2 | 2 | 2 | 2 | 4 | 4 | 2 | 2 | 2 | 2 | 2 | Duplication of CORT0A010140 |
| cort_c_1:2738000..2740000 | 2 | 2 | 1 | 2 | 2 | 1 | 1 | 2 | 2 | 2 | 2 | 2 | 2 | 2 | 2 | 2 | 2 | 2 | 2 | 2 | 2 | 1 | 1 | 2 | 2 | 2 | 0 | 2 | Region surrounded by gaps |
| cort_c_2:184000..186000 | 2 | 12 | 22 | 2 | 2 | 2 | 2 | 2 | 2 | 2 | 2 | 2 | 2 | 2 | 2 | 2 | 2 | 2 | 2 | 2 | 2 | 2 | 2 | 2 | 2 | 2 | 2 | 2 | Amplification of CORT0B00910 |
| cort_c_2:523000..524000 | 2 | 2 | 2 | 1 | 1 | 1 | 1 | 1 | 1 | 1 | 1 | 2 | 2 | 2 | 2 | 2 | 2 | 2 | 2 | 2 | 2 | 1 | 1 | 0 | 0 | 0 | 2 | 2 | Intein in CORT0B02480 9THS1) |
| cort_c_2:805000..807000 | 2 | 2 | 2 | 2 | 2 | 0 | 0 | 2 | 0 | 2 | 1 | 2 | 2 | 2 | 2 | 2 | 2 | 2 | 2 | 2 | 2 | 1 | 2 | 1 | 1 | 1 | 2 | 2 | Overlapping CORT0B03700 |
| cort_c_2:836000..843000 | 2 | 2 | 2 | 1 | 1 | 2 | 2 | 2 | 1 | 2 | 2 | 2 | 2 | 2 | 2 | 2 | 2 | 2 | 2 | 2 | 2 | 2 | 2 | 2 | 2 | 2 | 1 | 1 | Complex, SAP gene cluster. |
| cort_c_2:859000..863000 | 6 | 6 | 6 | 2 | 2 | 2 | 2 | 3 | 2 | 3 | 2 | 3 | 3 | 3 | 4 | 3 | 4 | 3 | 4 | 3 | 3 | 2 | 2 | 2 | 2 | 3 | 2 | 2 | Copy number increase of gene CORT0B03950 |
| cort_c_2:973000..974000 | 0 | 0 | 0 | 1 | 1 | 1 | 2 | 1 | 1 | 1 | 0 | 1 | 1 | 0 | 1 | 1 | 1 | 2 | 1 | 2 | 1 | 2 | 2 | 1 | 1 | 1 | 2 | 2 | Large heterozygous inter-genic deletion between CORT0B04510 and CORT0B04520 |
| cort_c_2:992000..996000 | 1 | 1 | 1 | 1 | 2 | 2 | 2 | 0 | 2 | 0 | 2 | 2 | 2 | 1 | 2 | 2 | 2 | 2 | 2 | 2 | 2 | 2 | 2 | 2 | 2 | 2 | 2 | 2 | Near gaps in reference genome |
| cort_c_2:1246000..1249000 | 1 | ~40 | ~22 | ~18 | 2 | 2 | 2 | ~20 | 2 | ~20 | 2 | ~20 | ~20 | 2 | ~20 | 2 | ~20 | ~20 | ~12 | ~20 | ~12 | 2 | 2 | 2 | 2 | 2 | 2 | 2 | Various CNVs inside region; major amplication of RTA3/2 (CORT0B05830/5840) |
| cort_c_2:1975000..1977000 | 2 | 2 | 2 | 2 | 2 | 2 | 2 | 2 | 2 | 2 | 2 | 2 | 2 | 2 | 2 | 2 | 2 | 2 | 2 | 2 | 2 | 2 | 2 | 2 | 2 | 3 | 2 | 2 | Coverage increase in 498, adjacent to gap in reference |
| cort_c_3:65000..66000 | 0 | 0 | 0 | 0 | 0 | 0 | 0 | 0 | 0 | 0 | 0 | 1 | 1 | 1 | 1 | 1 | 1 | 1 | 1 | 1 | 1 | 0 | 0 | 2 | 2 | 0 | 2 | 2 | Heterozygous inter-genic deletion between CORT0C00390 and CORT0C00400 |
| cort_c_3:286000..289000 | 1 | 1 | 1 | 2 | 1 | 1 | 1 | 1 | 1 | 1 | 2 | 2 | 2 | 2 | 2 | 2 | 2 | 2 | 2 | 2 | 2 | 2 | 2 | 2 | 2 | 2 | 2 | 2 | Region overlapping CORT0C01410 |
| cort_c_3:427000..431000 | 0 | 2 | 1 | 1 | 1 | 0 | 2 | 1 | 1 | 1 | 1 | 1 | 1 | 1 | 1 | 1 | 2 | 1 | 2 | 1 | 2 | 1 | 2 | 0 | 0 | 2 | 0 | 2 | Region overlapping CORT0C01980, CORT0C01990 and CORT0C02000 |
| cort_c_3:599000..610000 | 0 | 0 | 0 | 2 | 2 | 2 | 2 | 2 | 2 | 2 | 2 | 2 | 2 | 2 | 2 | 2 | 2 | 2 | 2 | 2 | 2 | 2 | 2 | 2 | 2 | 2 | 0 | 2 | Adjacent to IFF gene. |
| cort_c_3:955000..963000 | 1 | 1 | 1 | 2 | 2 | 1 | 1 | 2 | 2 | 2 | 2 | 2 | 2 | 1 | 1 | 2 | 1 | 2 | 0 | 2 | 1 | 2 | 2 | 2 | 2 | 2 | 2 | 2 | 7.3kb deletion in parent B, deletion of ALS gene CORT0C04210 |
| cort_c_3:1480000..1483000 | 2 | 2 | 2 | 0 | 0 | 0 | 0 | 0 | 0 | 0 | 0 | 2 | 2 | 1 | 1 | 2 | 1 | 2 | 0 | 2 | 0 | 0 | 0 | 0 | 0 | 1 | 2 | 2 | 2.7kb deletion in parent B |
| cort_c_3:1553000..1555000 | 1 | 1 | 1 | 2 | 2 | 2 | 2 | 2 | 2 | 2 | 2 | 2 | 2 | 1 | 1 | 2 | 1 | 2 | 0 | 1 | 0 | 4 | 2 | 2 | 2 | 2 | 0 | 2 | Adjacent to gap in reference genome |
| cort_c_3:1642000..1645000 | 2 | 2 | 2 | 2 | 2 | 2 | 2 | 2 | 2 | 2 | 2 | 2 | 2 | 2 | 2 | 2 | 2 | 2 | 2 | 2 | 2 | 0 | 2 | 2 | 2 | 1 | 2 | 2 | Region near telomere |
| cort_c_4:1000..3000 | 0 | 0 | 0 | 0 | 0 | 0 | 0 | 0 | 0 | 0 | 0 | 2 | 2 | 2 | 2 | 2 | 2 | 2 | 2 | 2 | 2 | 0* | 0** | 0 | 0 | 0** | 2 | 2 | Region near telomere. *748 retained first 670 bases. **424 and 498 have an extended deletion until 3.4kb. |
| cort_c_4:547000..552000 | ND | ND | ND | ND | ND | 2 | ND | 2 | ND | 2 | ND | 2 | 2 | 2 | 2 | 2 | 2 | 2 | 2 | 2 | 2 | ND | ND | ND | ND | 2 | 2 | 2 | Triple tandem repeat for genes CORT0D02950, CORT0D02960 and CORT0D02970 in reference genome. B allele appears to have fewer repeats. |
| cort_c_4:835000..836000 | 2 | 2 | 2 | 0 | 0 | 0 | 0 | 0 | 0 | 0 | 0 | 2 | 2 | 2 | 2 | 2 | 2 | 2 | 2 | 2 | 2 | 2 | 2 | 2 | 2 | 2 | 0 | 2 | Region is between gaps |
| cort_c_4:891000..892000 | 0 | 0 | 0 | 1 | 1 | 1 | 1 | 0 | 2 | 0 | 1 | 0 | 0 | 0 | 0 | 0 | 0 | 0 | 0 | 0 | 0 | 0 | 0 | 0 | 0 | 1 | 0 | 0 | Four highly similar copies of CORT0D04520 (from PacBio assembly). Reference genome contains only three copies, one of which is missassembled. One allele of 498 has only two copies of the gene |
| cort_c_4:1074000..1075000 | 0 | 0 | 0 | 0 | 0 | 0 | 0 | 0 | 0 | 0 | 0 | 1 | 1 | 1 | 1 | 1 | 1 | 0 | 1 | 0 | 2 | 1 | 1 | 2 | 2 | 1 | 2 | 2 | Heterozygous intergenic deletion. |
| cort_c_5:155000..158000 | 2 | 2 | 2 | 2 | 1 | 2 | 2 | 1 | 2 | 2 | 1 | 2 | 2 | 2 | 2 | 2 | 1 | 2 | 2 | 2 | 2 | 1 | 1 | 1 | 1 | 0 | 0 | 2 | Tandem duplication, adjacent CNVs can not be scored |
| cort_c_5:316000..319000 | 0 | 0 | 0 | 0 | 0 | 0 | 0 | 0 | 0 | 0 | 0 | 0 | 0 | 0 | 0 | 0 | 0 | 0 | 0 | 0 | 0 | 1 | 1 | 1 | 1 | 1 | 0* | 2 | Large deletion which includes gene CORT0E01520. *428 contains shorter deletion |
| cort_c_5:399000..400000 | 2 | 2 | 2 | 2 | 2 | 2 | 2 | 2 | 2 | 2 | 2 | 4 | 4 | 4 | 4 | 4 | 4 | 4 | 4 | 4 | 4 | 2 | 2 | 2 | 2 | 2 | 2 | 2 | Amplified region including gene CORT0E01870 |
| cort_c_5:911000..914000 | 2 | 2 | 2 | 2 | 2 | 2 | 2 | 2 | 2 | 2 | 2 | 2 | 2 | 2 | 2 | 2 | 2 | 2 | 2 | 2 | 2 | 1 | 1 | 1 | 1 | 0 | 2 | 2 | Deletion of CORT0E04080 and CORT0E04090 |
| cort_c_6:1000..7000 | 2 | 2 | 2 | 2 | 2 | 2 | 2 | 2 | 2 | 2 | 2 | 2 | 2 | 2 | 2 | 2 | 2 | 2 | 2 | 2 | 2 | 2 | 2 | 2 | 2 | 1 | 2 | 2 | Heterozygous deletion in 498 of first 6.8kb, near telomere |
| cort_c_6:73000..75000 | 2 | 2 | 2 | 0 | 0 | 0 | 0 | 0 | 0 | 0 | 0 | 2 | 2 | 2 | 2 | 2 | 2 | 2 | 2 | 2 | 2 | 2 | 2 | 2 | 2 | 2 | 2 | 2 | Deletion of one gene in tandem duplication ( CORT0F00560 and CORT0F00570). Specific to clade 2 strains |
| cort_c_6:142000..147000 | ND | ND | ND | ND | ND | ND | ND | ND | ND | ND | ND | ND | ND | ND | ND | ND | ND | ND | ND | ND | ND | ND | ND | ND | ND | ND | ND | ND | Complex variations, around metallothionein (CORT0F00874, CORT0F00877) and SAP gene (CORT0F00880) |
| cort_c_6:308000..310000 | 0 | 0 | 0 | 1 | 1 | 1 | 2 | 1 | 2 | 1 | 0 | 1 | 1 | 1 | 1 | 1 | 1 | 1 | 1 | 1 | 1 | 1 | 1 | 2 | 2 | 1 | 2 | 2 | Region with low coverage in 90-125 |
| cort_c_6:563000..564000 | 2 | 2 | 2 | 2 | 3 | 4 | 3 | 2 | 4 | 2 | 6 | 3 | 3 | 3 | 3 | 3 | 3 | 3 | 3 | 3 | 3 | 3 | 3 | 3 | 3 | 3 | 2 | 2 | Allelic differences for CORT0F02740. Possible additional copy in B allele |
| cort_c_7:1000..11000 |  |  |  |  |  |  |  |  |  |  |  |  |  |  |  |  |  |  |  |  |  |  |  |  |  |  |  |  | Multiple differences near telomeres |
| cort_c_7:727000..729000 | 1 | 2 | 1 | 0 | 0 | 0 | 0 | 0 | 0 | 0 | 0 | 0 | 0 | 0 | 0 | 0 | 0 | 0 | 0 | 0 | 0 | 2 | 1 | 1 | 1 | 1 | 0 | 2 | Deletion of CORT0G03560 |
| cort_c_7:836000..837000 | 2 | 2 | 2 | 2 | 2 | 2 | 2 | 2 | 2 | 2 | 2 | 2 | 2 | 2 | 2 | 2 | 2 | 2 | 2 | 2 | 2 | 2 | 2 | 2 | 2 | 2 | 0 | 2 | Deletion of CORT0G04060 in 428 |
| cort_c_7:934000..938000 | 2 | 2 | 2 | 2 | 2 | 2 | 2 | 2 | 2 | 2 | 2 | 0 | 0 | 0 | 0 | 0 | 0 | 0 | 0 | 0 | 0 | 2 | 2 | 2 | 2 | 1 | 2 | 2 | 3.8kb deletion in clade 3 |
| cort_c_8:11000..12000 | 0 | 0 | 0 | 1 | 1 | 1 | 1 | 2 | 2 | 1 | 0 | 2 | 2 | 2 | 2 | 2 | 2 | 2 | 2 | 2 | 2 | 2 | 2 | 2 | 2 | 2 | 0 | 2 | Deletion of intergenic reagion |
| cort_c_8:12000..13000 | 2 | 2 | 2 | 1 | 1 | 1 | 1 | 0 | 0 | 1 | 2 | 2 | 2 | 2 | 2 | 2 | 2 | 2 | 2 | 2 | 2 | 2 | 2 | 2 | 2 | 2 | 2 | 2 | Multiple sequence differences across clades overlapping and downstream of CORT0H00180 (CSA1) |
| cort_c_8:125000..126000 | 2 | 2 | 2 | 1 | 2 | 2 | 2 | 2 | 2 | 2 | 0 | 2 | 2 | 2 | 2 | 2 | 2 | 2 | 2 | 2 | 2 | 2 | 2 | 2 | 2 | 2 | 2 | 2 | Deletion spanning CORT0H00780 (TPO2) and CORT0H00770 |
| cort_c_8:288000..289000 | 0 | 0 | 0 | 1 | 1 | 1 | 1 | 1 | 1 | 1 | 1 | 1 | 1 | 1 | 1 | 1 | 1 | 1 | 1 | 1 | 1 | 2 | 2 | 2 | 2 | 2 | 0 | 2 | Deletion (homozygous in clade 1 and 428, heterozygous in clade 2 and 3). |
| cort_c_8:322000..328000 | 2 | 2 | 2 | 2 | 2 | 2 | 2 | 2 | 2 | 2 | 2 | 2 | 2 | 2 | 2 | 2 | 1 | 2 | 2 | 2 | 2 | 2 | 2 | 2 | 2 | 2 | 2 | 2 | 7kb heterozygous deletion in 599. Surrounded by gaps |
| cort_c_8:522000..524000 | 2 | 2 | 2 | 1 | 0 | 2 | 1 | 2 | 2 | 2 | 2 | 1 | 2 | 1 | 0 | 1 | 0 | 1 | 0 | 1 | 0 | 1 | 1 | 1 | 1 | 1 | 2 | 2 | 3.65kb deletion |
| cort_c_8:607000..613000 | 8 | 12 | 6 | 2 | 2 | 2 | 2 | 2 | 2 | 2 | 2 | 2 | 2 | 2 | 2 | 2 | 2 | 2 | 2 | 2 | 2 | 2 | 2 | 2 | 2 | 2 | 2 | 2 | Coverage increase in clade 1 samples near telomere, around sodium transporter CORT0H02780 |
